# New insights into light spectral quality inhibits the plasticity elongation of maize mesocotyl and coleoptile during seed germination

**DOI:** 10.3389/fpls.2023.1152399

**Published:** 2023-03-15

**Authors:** Xiaoqiang Zhao, Yining Niu, Zakir Hossain, Bingyu Zhao, Xiaodong Bai, Taotao Mao

**Affiliations:** ^1^ State Key Laboratory of Aridland Crop Science/College of Agronomy, Gansu Agricultural University, Lanzhou, China; ^2^ Swift Current Research and Development Centre, Agriculture and Agri-Food Canada, Swift Current, SK, Canada; ^3^ School of Plant and Environmental Sciences, College of Agriculture and Life Sciences, Blacksburg, VA, United States

**Keywords:** maize mesocotyl/coleoptile, light spectral quality, RNA-sequencing, WGCNA, circadian rhythm, phytohormones, cytoskeleton, lignin metabolism

## Abstract

The plastic elongation of mesocotyl (MES) and coleoptile (COL), which can be repressed by light exposure, plays a vital role in maize seedling emergence and establishment under adverse environmental conditions. Understanding the molecular mechanisms of light-mediated repression of MES and COL elongation in maize will allow us to develop new strategies for genetic improvement of these two crucial traits in maize. A maize variety, Zheng58, was used to monitor the transcriptome and physiological changes in MES and COL in response to darkness, as well as red, blue, and white light. The elongation of MES and COL was significantly inhibited by light spectral quality in this order: blue light > red light > white light. Physiological analyses revealed that light-mediated inhibition of maize MES and COL elongation was closely related to the dynamics of phytohormones accumulation and lignin deposition in these tissues. In response to light exposure, the levels of indole-3-acetic acid, trans-zeatin, gibberellin 3, and abscisic acid levels significantly decreased in MES and COL; by contrast, the levels of jasmonic acid, salicylic acid, lignin, phenylalanine ammonia-lyase, and peroxidase enzyme activity significantly increased. Transcriptome analysis revealed multiple differentially expressed genes (DEGs) involved in circadian rhythm, phytohormone biosynthesis and signal transduction, cytoskeleton and cell wall organization, lignin biosynthesis, and starch and sucrose metabolism. These DEGs exhibited synergistic and antagonistic interactions, forming a complex network that regulated the light-mediated inhibition of MES and COL elongation. Additionally, gene co-expression network analysis revealed that 49 hub genes in one and 19 hub genes in two modules were significantly associated with the elongation plasticity of COL and MES, respectively. These findings enhance our knowledge of the light-regulated elongation mechanisms of MES and COL, and provide a theoretical foundation for developing elite maize varieties with improved abiotic stress resistance.

## Introduction

Maize (*Zea mays* L.), a major multipurpose crop grown for food, feed, and biofuel industry, is extremely susceptible to environmental perturbations (Nguy-Robertson et al., 2015; Liu et al., 2020). The stability of yield and sustainability of production in maize are directly related to the social economy and livelihood of a community (http://faostat.fao.org/). In long-term agricultural practice, astute observers have noticed an interesting phenomenon, whereby the mesocotyl (MES) and coleoptile (COL) of maize are synergistically elongated during seed germination, and maize seedlings with longer MES and COL grow better in deep soil layers and respond positively to various abiotic and biotic constraints. For example, the abundant evaporation and scarce precipitation in the arid and semi-arid regions of the Southwest USA, Western Mexico, and Northwest China, the seeds of maize varieties P1213733 (Komona) (Troyer, 1997) and 40107 (Zhao and Niu, 2022) are widely sown at a depth of ≥25 cm in these regions, where they are able to obtain sufficient water for rapid germination, ensuring normal seedling growth. Similarly, elite varieties of wheat (*Triticum aestivum* L.) Moro (Schillinger et al., 1998) and Hong-mang-mai (Takahashi et al., 2001), are often planted at a depth of 10 cm beneath soil surface in low precipitation areas of the Inland Pacific Northwest and Loess Plateau of China due to their excellent emergence. Both the MES and COL tissues of maize are sensitive to cold injury during seed germination (Zhao et al., 2022b). Since the temperature of deep soil layers is higher in early spring, it facilitates seed germination and prevents cold-mediated damage to MES and COL (Zhao and Niu, 2022). In addition, the deep-seeding strategy improves maize yield of reducing bird pecking (Shi et al., 2022), rodent infestation (Brown et al., 2003), soil-borne fungal invasions (Niu et al., 2020), and pre-emergent Herbicide-associated phytotoxicity (Brown et al., 2003). Thus, unravelling the molecular mechanism of cooperative elongation between MES and COL in maize under different environmental conditions is important for improving early seedling establishment in adverse environments and will be pertinent not only to maize breeding programs but also to the management of maize populations.

Generally, soon after the maize seeds germinated in the soil, the elongated MES (an embryonic structure between COL node and basal part of seminal root in seedlings) pushes the COL (a conical structure that sheaths the shoot apex) through the soil surface. At the soil surface, incident light represses MES and COL elongation, induces leaf expansion, and promotes root formation (Liu et al., 2017; Rodríguez and Cassab, 2021; Zhao and Zhong, 2021). As cells are recruited to the emerging leaf primordia, proplastids differentiate into the dimorphic bundle sheath and mesophyll cell chloroplasts, and the photoautotrophic phase of sporophytic development is initiated (Markelz et al., 2003). In higher plants, the phytochromes (PHYs; red/far-red light photoreceptors), blue light and UV-A-absorbing cryptochromes (CRYs), and UV-B photoreceptors enable the developing seedlings to monitor the spectral quality, flux, direction, and duration of incident light, which regulate the circadian clock, light-dependent seed germination, seedling de-etiolation, chloroplast differentiation, and organ development (Kevei and Nagy, 2003). Overexpression of *cryptochrome-1* gene, i.e., *OsCRY1a* (AB073546) or *OsCRY1b* (AB073547) in rice (*Oryza sativa* L.) induce blue light perception, leading to the suppression of COL and leaf sheath elongation (Hirose et al., 2006). This phenomenon is closely associated with the repression of a *gibberellin 3-oxidase 2* (*OsGA3ox2*) gene in light signaling (Hirose et al., 2012). Analysis of multiple maize accessions of temperate and tropical/semitropical origins, together with teosinte (*Zea mays* ssp*. Parviglumis*), reveals that end-of-day far-red light (EOD-FR)-mediated MES responses have not been lost during the domestication or breeding process, and EOD-FR reduces abscisic acid (ABA) levels in the MES of both the wild-type and *phyb1 phyb2* double mutant plants (Dubois et al., 2010).

Previous studies confirmed that multiple phytohormones, including indole-3-acetic acid (IAA), gibberellin 3 (GA_3_), brassinosteroid (BR), and ABA, play crucial roles in regulating the cooperative elongation of MES and COL in maize under deep-seeding stress (Zhao et al., 2021a; Zhao and Niu, 2022). Exogenous IAA-induced (10^-6^ to 10^-4^ M) (Zhao and Wang, 2010), GA_3_-induced (10^-6^ to 10^-5^ M) (Zhao et al., 2010), and 24-epibrassinolide (EBR)-induced (4.16×10^-3^ M) (Zhao et al., 2021a) MES and COL elongation result in improving maize deep-seeding tolerance. Walton and Ray (1981) showed that the auxin (AUX)-binding activity localized on endoplasmic reticulum membranes and elongation response along with the MES length decreased in maize after 4 h in red light environments. Studying loss-of-function mutants of *ABP1* and *ABP4* genes (encoding AUX-binding proteins), Jana and Martin (2012) demonstrated that *ABP1* and *ABP4* genes are involved in the light- and AUX-induced down-regulation of *PHYB* transcript levels in maize MES, and suggested a cross-talk between AUX and light signaling. Hamada et al. (1994) observed that propyzamide could disrupt cortical microtubules in cells in the upper regions of maize MES in GA_3_-untreated seedlings and caused swelling of the cells. The KTN80 (p80 subunit of microtubule-severing enzyme katanin) precisely regulates microtubule and reorients upon blue light illumination (Lindeboom et al., 2013), and subsequently changes the growth direction of hypocotyl cells in *Arabidopsis* (Wang et al., 2017). In addition, light-induced lignification in primary walls of maize seedlings result in cell-wall stiffening and subsequent inhibition of cell elongation process in the MES elongation zone (Schopfer et al., 2001).

Unlike deep-seeding stress, increasing observations suggest that varying light quality can significantly inhibit plasticity elongation of MES and COL during maize germination, indicating the existence of a complex control mechanism for light quality-inhibited MES and COL elongation in maize seedlings (Walton and Ray, 1981; Jones et al., 1991; Schopfer et al., 2001; Markelz et al., 2003; Dubois et al., 2010; Kusnetsov et al., 2020). However, it remains to be determined how precisely the control mechanism is triggered in MES and COL under different light quality stimulations. Hence, we used high-throughput RNA sequencing (RNA-Seq), liquid chromatography-tandem mass spectrometry (UHPLC-MS/MS), and quantitative real-time PCR (qRT-PCR) to i) further investigate the molecular basis and physiological responses of plasticity elongation between MES and COL in Zheng58 seedlings under red, blue, and white light conditions, ii) explore the gene co-expression modules and genes that act as critical network hubs, and iii) interpret the biological processes and pathways associated with MES and COL development. These results will provide molecular resources for light-induced plasticity elongation of MES and COL during maize germination, and establish a theoretical foundation for the development of longer MES and COL germplasms. Therefore, it has important practical significance for improving maize survival strategies under severe environmental stresses.

## Materials and methods

### Plant materials and light treatments

The maize cv. Zheng58 is a representative inbred line, derives from the Reid heterotic group, with superior drought tolerance (Wang et al., 2022). The seeds of cv. Zheng58 were first sterilized with 0.5% (v/v) sodium hypochlorite solution for 10 min, rinsed three times with double-distilled water, and soaked in double-distilled water for 24 h in darkness. After 20 soaked seeds were pre-cultured in the germinating box for five days in darkness at a 22 ± 0.5°C constant temperature, the etiolated seedlings were placed into plant chambers and illuminated with various light-emitting diode (LED) lamps, including red light (peak wavelength: 660 nm; photosynthetic photon flux density (PFD): 22 μM m^-2^ s^-1^), blue light (peak wavelength: 450 nm; PFD: 13 μM m^-2^ s^-1^), and white light (PFD 17 μM m^-2^ s^-1^) in each chamber (Yang et al., 2016). The seedlings were cultured with 12 h light per day, 22 ± 0.5°C constant temperature, and 70% relative humidity for five days, and which were watered with 20 mL sterile Hoagland solution per pot at 2-day intervals, while the control seedlings was cultured in darkness. The tissues of MES and COL were then separated, frozen in liquid nitrogen immediately, and stored at -80°C for physiological measurements, RNA extraction, and gene expression analysis.

### Growth and physiology parameters measurement

After the seedlings were cultured for five days in four light conditions, we measured mesocotyl length (MESL), coleoptile length (COLL), total length of mesocotyl and coleoptile (MESL+COLL), ratio of mesocotyl length to coleoptile length (MESL/COLL), seedling length (SDL), root length (RL), mesocotyl coarse (MESC), coleoptile coarse (COLC), ratio of mesocotyl coarse to coleoptile coarse (MESC/COLC), seedling stem diameter (SD), root coarse (RC), mesocotyl fresh weight (MESW), coleoptile fresh weight (COLW), ratio of mesocotyl fresh weight to coleoptile fresh weight (MESW/COLW), seedling fresh weight (SW), root fresh weight (RW), root to shoot ratio (RSR), and root number (RN), respectively (Zhao et al., 2021a; Zhao et al., 2022a).

Frozen MES or COL tissue was used to assay the content of IAA, trans-zeatin (tZ), GA_3_, ABA, jasmonic acid (JA), and salicylic acid (SA), respectively (Zhao et al., 2021b). Briefly, 0.5 g sample was ground in liquid nitrogen and digested in 5 mL methanol-formic acid solution (99:1, v:v) for 12 h at 4°C, centrifuged at 12,000 rpm at 4°C for 20 min to remove debris. The supernatant was collected, and the above procedure was repeated one or more times. After pigment removed by Cleanert ODS C18 solid phase extraction column (Tianjin Aiger Co., Ltd., China), the liquid was dried by nitrogen flow at 25°C, dissolved using 1 mL methanol, and filtered with a 0.22 μM membrane filter. Finally, the supernatant was transferred to a vial for UHPLC-MS/MS analysis. The standards of IAA (CAS: 87-51-4), tZ (CAS: 1637-39-4), GA_3_ (CAS: 77-065-5), ABA (CAS: 21293-29-8), JA (CAS: 7706-92-7), and SA (CAS: 69-72-7) were purchased from Shanghai Yuanye Bio-Technology Co., Ltd., Shanghai, China.

The phenylalanine ammonia-lyase (PAL) and peroxidase (POD) are important regulatory enzymes for lignin biosynthesis (Zhao et al., 2022a). To evaluate the PAL activity of MES or COL tissue, 0.5 g sample was homogenized in 5 mL of 25 mM borate buffer, pH 8.8 containing 2 μL β-mercaptoethanol, and a pinch of polyvinyl polypyrrolidone (PVP); the homogenate was filtered through pumping air and centrifuged at 12,000 rpm at 4°C for 10 min (Gholizadeh and Kohnehrouz, 2010). The supernatant was then stored at 4°C and PAL activity of MES or COL was measured by monitoring changes in absorbance values at 290 nm (Zhao and Zhong, 2021). To measure POD activity of MES or COL tissue, 0.5 g sample was homogenized in 1 mL of 50 mM ice-cold potassium-phosphate buffer, pH 7.0 containing 100 mM potassium chloride, 1 mM ascorbate, 5 mM β- mercaptoethanol, and 10% glycerol (w/v) using a pre-cooled mortar and pestle; the homogenate was centrifuged at 12,000 rpm at 4°C for 10 min. The supernatant was then stored at 4°C and POD activity of MES or COL was measured by monitoring changes in absorbance values at 470 nm (Zhao et al., 2021b). For lignin analysis, 0.5 g MES or COL tissue sample was homogenized in 5 mL of 95% ethanol (v/v) and centrifuged at 12,000 rpm at 4°C for 10 min; the sediment was rinsed three times with ethanol-n-hexane solution (1:1, v:v) and dried. The pellet was dissolved in 0.5 mL bromide acetyl-glacial acetic acid solution (1:3, v:v) and incubated at 70°C for 30 min in a water bath. The reaction was terminated by adding 0.9 mL 2 M NaOH, mixed with 5 mL glacial acetic acid and 0.1 mL 7.5 M hydroxylamine hydrochloride, and centrifuged at 4,500 rpm for 5 min. The supernatant was used for assaying the lignin content of MES or COL by monitoring changes in absorbance value at 280 nm (Zhao et al., 2021b).

Data of growth and physiology parameters was performed by Duncan’s multiple range test (*p*<0.05) using the IBM-SPSS Statistics v.19.0 (SPSS Inc.) (https://www.ibm.com/products/spss-statistics). The Pearson pairwise correlation analysis was performed using the Genescloud tool, a free online platform for data analysis (https://www.genescloud.cn).

### RNA sample collection and illumina sequencing

Total RNA samples from MES and COL of Zheng58 seedlings under red, blue, and white light conditions and darkness, were extracted using the TRIZOL reagent (Invitrogen, Carlsbad, CA, United States) following the manufacturer’s protocol. RNA-Seq was then constructed using Illumina NovaSeq PE150 Sequencer at Nanjing Genepioneer Biotechnologies Company, Nanjing, China. After filtering, clean sequence reads were aligned to Zea_mays B73_V4 reference genome using HISAT v.2.2.1. Fragments per kilobase of transcript per million mapped read (FPKM) values were estimated using Cufflinks v.2.2.1. The transformed and normalized gene expression values with log2 (FPKM+1) were used for principal component analysis (PCA). PCA was performed using the fast.prcomp function from gmodels in R 4.0.1.

### Differentially expressed genes identification and functional analyses

Differential expression analysis was conducted using the DESeq R in Bioconductor (http://www.bioconductor.org/). In each pairwise comparison, the DEGs were identified as having FDR (the Benjamini and Hochberg false discovery rate) < 0.05, FPKM > 1, and |log_2_ fold change (FC)| > 1. Further analysis of DEGs, including Gene Ontology (GO) enrichment analysis, Clusters of Orthologous Groups of Proteins (COG) analysis, Kyoto Encyclopedia of Genes and Genomes (KEGG) analysis, and NCBI non-redundant protein sequences (Nr) annotation, were performed.

### Weighted gene co-expression network analysis

Gene co-expression modules were constructed with R package WGCNA v1.68 (Los Angeles, CA, USA) (Langfelder and Horvath, 2008). To identify plasticity elongation of MES and COL associated modules under four light conditions, we correlated module eigengenes with 24 traits in all light treatments and drew their correlation heat maps. For each of the tissue, the genes with mean FPKM > 1 for the 12 samples were analyzed. For which the soft threshold power β was set as nine, and mergeCutHeight = 0.4 was used to merge similar modules. A module was defined as significant if the *p*-value for module-trait association was 0.05 (Wang et al., 2022). The OmicShare tool2 (https://www.omicshare.com/) was used to map the network visualization of genes within the module, and genes with high co-expression connection within the module were filtered, and Cytoscape v.3.7.1 (Seattle, WA, USA) was used to visualize co-expression network.

### qRT-PCR analysis

Purified total RNA (0.5 μg) was reverse-transcribed to synthesize first-strand cDNA using HiScript^®^Q RT SuperMix for qPCR (Vazyme, China) according to the manufacturer’s protocol. qRT-PCR was conducted on quantum Studio 5 real-time PCR system (Thermo Fisher Scientific, MA, United States) using super real premix plus (SYBR Green) (Tiangen, Shanghai, China). The specific primers for 23 selected genes were designed with Quantprime qPCR primer design tool (https://quantprime.mpimp-golm.mpg.de) (**Supplementary Table 1**). The gene relative expression level was calculated by the 2^-△△Ct^ method, with *ZmActin1* (*Zm00001d010159*) as an internal reference gene (Zhao et al., 2022b).

## Results

### Phenotypic and physiological variations of maize seedlings in four light stimulations

**Figure 1:**
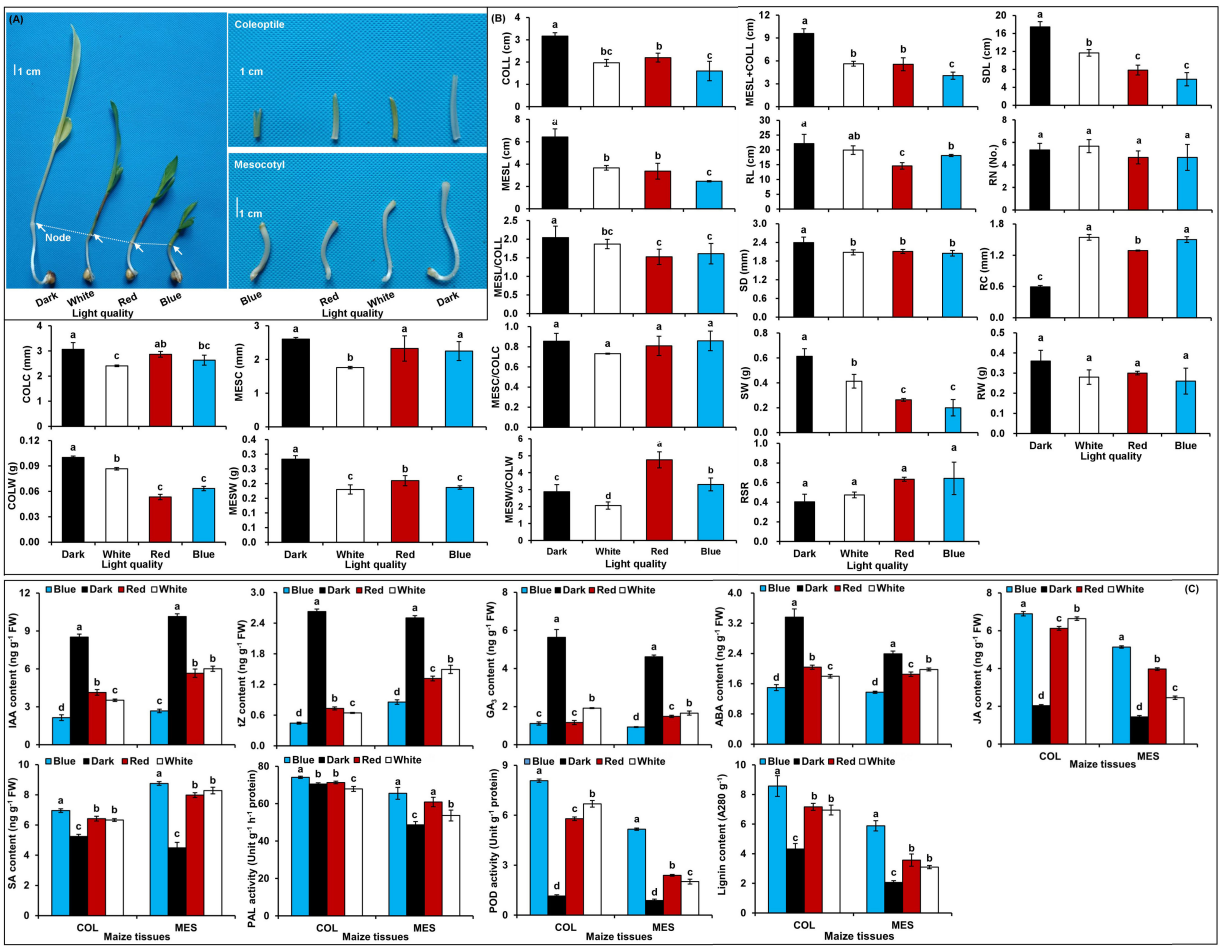
Growth characteristics and physiological changes in mesocotyl (MES) and coleoptile (COL) of Zheng58 seedlings in four light stimulations. Growth of mesocotyl (MES) and coleoptile (COL) **(A)**. Statistical analysis of multiple growth parameters including mesocotyl length (MESL), coleoptile length (COLL), total length of mesocotyl and coleoptile (MESL+COLL), ratio of mesocotyl length to coleoptile length (MESL/COLL), seedling length (SDL), root length (RL), mesocotyl coarse (MESC), coleoptile coarse (COLC), ratio of mesocotyl coarse to coleoptile coarse (MESC/COLC), seedling stem diameter (SD), root coarse (RC), mesocotyl fresh weight (MESW), coleoptile fresh weight (COLW), ratio of mesocotyl fresh weight to coleoptile fresh weight (MESW/COLW), seedling fresh weight (SW), root fresh weight (RW), root to shoot ratio (RSR), and root number (RN). Different lowercase letters in different light stimulations indicated a significant difference (*p*<0.05) **(B)**. Changes in different physiological parameters, including indole-3-acetic acid (IAA) content, trans-zeatin (tZ) content, gibberellin 3 (GA_3_) content, abscisic acid (ABA) content, jasmonic acid (JA) content, salicylic acid (SA) content, phenylalanine ammonia-lyase (PAL) activity, peroxidase (POD) activity, and lignin content of MES and COL. Different lowercase letters in different light conditions indicated a significant difference (*p*<0.05) **(C)**.

In comparison with the control treatment, the red, blue, and white light treatment induced a clear inhibition on the growth of MES and COL during Zheng58 germination (*p*<0.05) (**Figure 1A, B**): MESL decreased by 47.7, 61.7, and 43.0%, while COLL decreased by 30.5, 49.5, and 37.9% in red, blue, and white light conditions, respectively (**Figure 1B**). Their MESC decreased by 10.7, 13.7, and 32.5%, while COLC decreased by 6.4, 14.0, and 21.4%, respectively (**Figure 1B**). These data showed that the longitudinal and transverse elongation of MES and COL in maize is clearly inhibited by different light exposures resulting in a significant decrease of MESW and COLW. However, the impact of red, blue, and white light stimulations on growth or biomass inhibition varied among themselves (**Figure 1B**). In addition, red, blue, and white light also significantly inhibited root and seedling development of Zheng58 (*p*<0.05) (**Figure 1B**). However, how the plasticity elongation of various tissues, especially in the MES and COL of Zheng58 seedlings, differed between the four light treatments is unclear.

**Figure 2:**
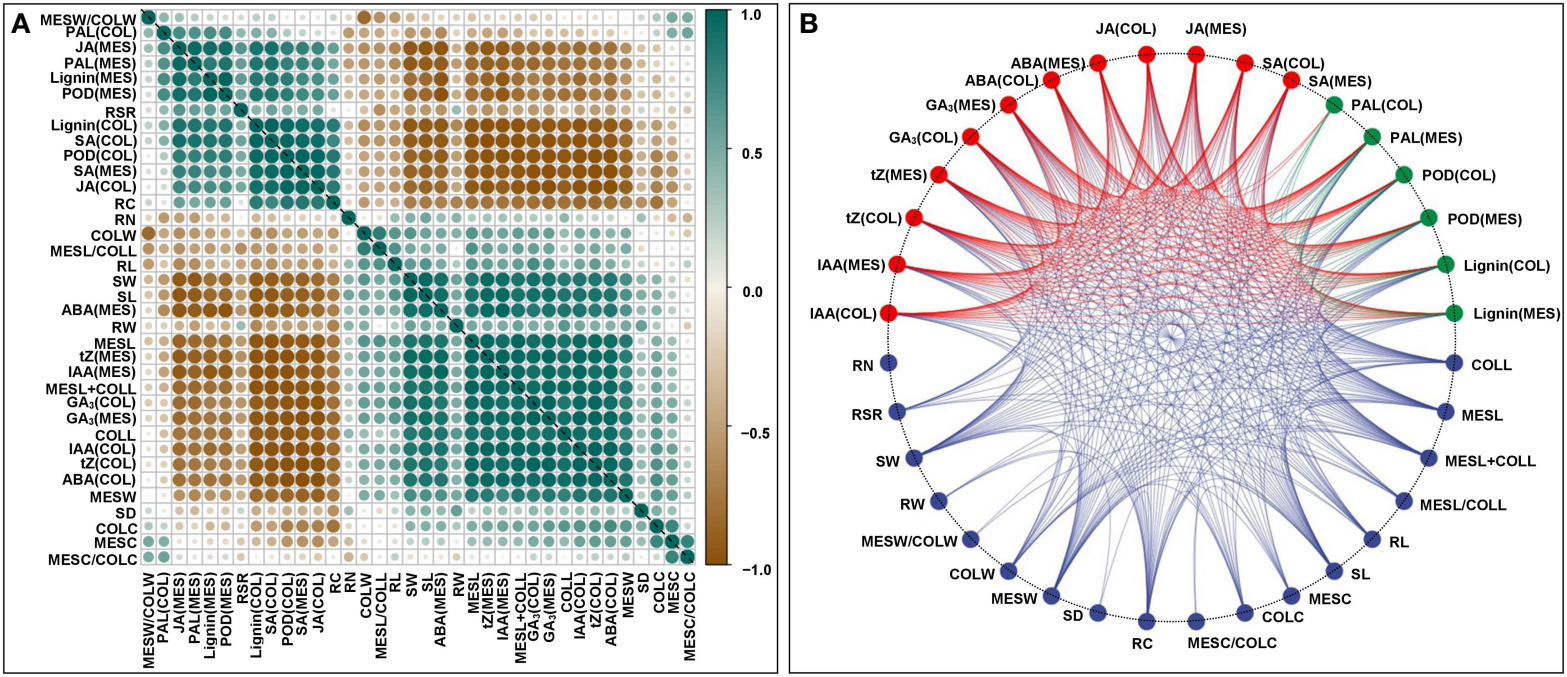
Framework of relations based on Pearson pairwise correlation analysis among 36 traits, including mesocotyl length (MESL), coleoptile length (COLL), total length of mesocotyl and coleoptile (MESL+COLL), ratio of mesocotyl length to coleoptile length (MESL/COLL), seedling length (SDL), root length (RL), mesocotyl coarse (MESC), coleoptile coarse (COLC), ratio of mesocotyl coarse to coleoptile coarse (MESC/COLC), seedling stem diameter (SD), root coarse (RC), mesocotyl fresh weight (MESW), coleoptile fresh weight (COLW), ratio of mesocotyl fresh weight to coleoptile fresh weight (MESW/COLW), seedling fresh weight (SW), root fresh weight (RW), root to shoot ratio (RSR), root number (RN), indole-3-acetic acid content in mesocotyl (MES)/coleoptile (COL) [IAA(MES/COL)], trans-zeatin content in MES/COL [tZ(MES/COL)], gibberellin 3 content in MES/COL [GA_3_(MES/COL)], abscisic acid content in MES/COL [ABA(MES/COL)], jasmonic acid content in MES/COL [JA(MES/COL)], salicylic acid content in MES/COL [SA(MES/COL)], phenylalanine ammonia-lyase activity in MES/COL [PAL(MES/COL)], peroxidase activity in MES/COL [POD(MES/COL)], lignin content in MES/COL [Lignin(MES/COL)] of Zheng58 seedlings in four light stimulations were performed by Genescloud tool (https://www.genescloud.cn). Pearson correlation coefficient diagram **(A)**. Interactive ring correlation diagram **(B)**.

To understand the physiological responses to plasticity elongation of MES and COL in Zheng58 seedlings, we measured the changes of six phytohormones levels, two enzymes activities, and lignin accumulations in both MES and COL under four light conditions (**Figure 1C**). Exposure to red, blue, and white light significantly decreased IAA, tZ, GA_3_, and ABA levels in both MES and COL compared to control treatment (**Figure 1C**). In contrast, JA, SA, and lignin contents significantly increased in both tissue types in response to different light exposure (**Figure 1C**). While POD activity increased significantly in both tissue types, and PAL activity increased clearly only in MES in Zheng58 seedlings because of light treatment (**Figure 1C**). To further explore the role of the phytohormones and lignin in maize MES and COL plasticity elongation, we conducted framework of relations based on Pearson pairwise correlation analysis among 36 tested traits in all four light stimulations. The correlation analysis predicted 351 groups with significant (*p*<0.01 or *p*<0.05) correlation between both tissue types (**Figures 2A, B**). From physiological changes and Pearson correlation analysis, it is likely that the phytohormones synthesis, transport, and signal transduction, as well as lignin biosynthesis and degradation could be involved in the plasticity elongation of maize MES and COL by irradiation to different spectra.

### Quality assessment of RNA-Seq data

**Figure 3:**
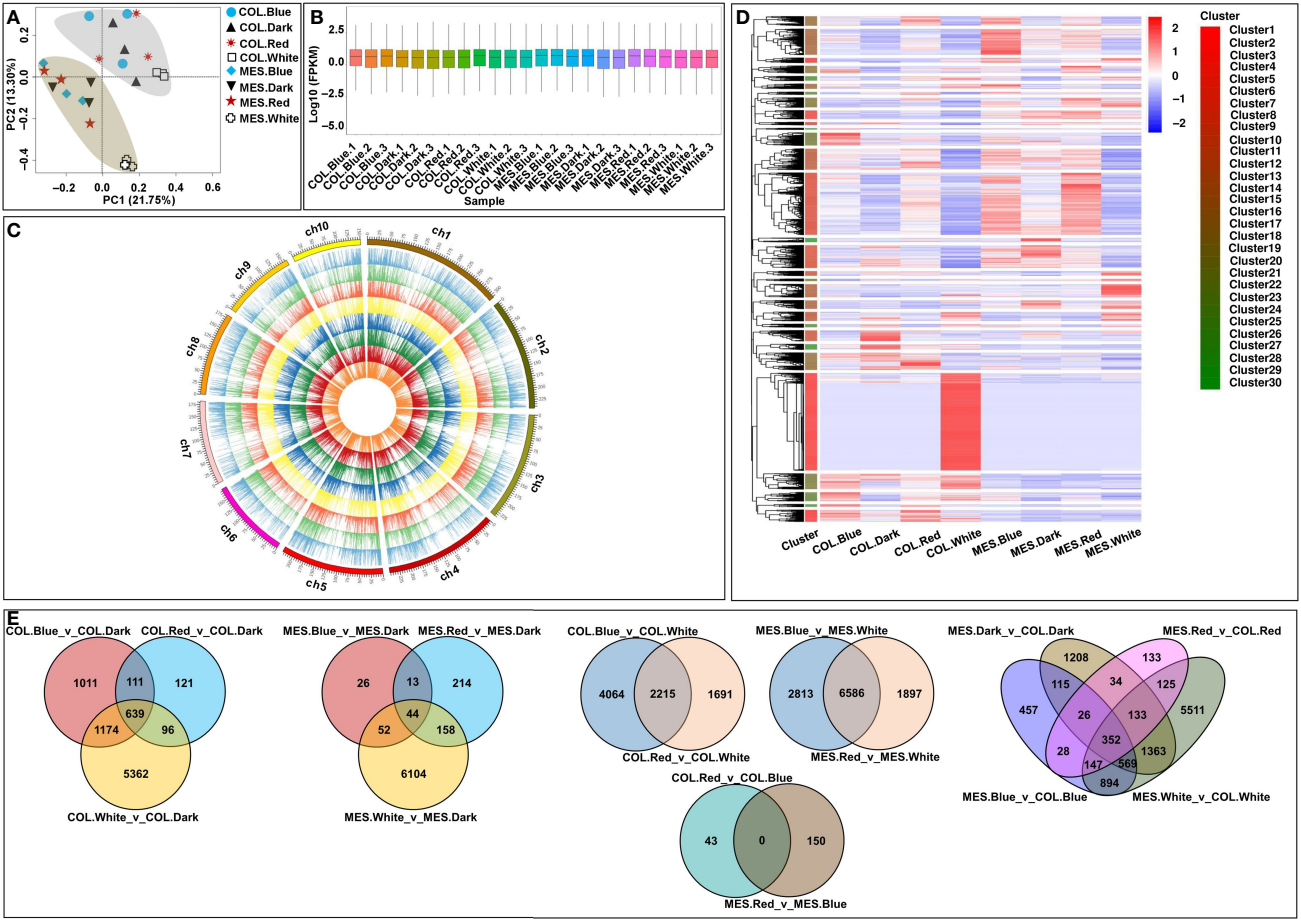
Global transcriptome sequencing and differentially expressed genes (DEGs) in mesocotyl (MES) and coleoptiple (COL) of Zheng58 seedlings in four light stimulations. Principal component analysis (PCA) of RNA-sequencing (RNA-Seq) data **(A)**. Gene expression of all samples, the boxplots with different colors indicating different samples analyzed at regular intervals **(B)**. Chromosome-wise distribution and expression profiles of all expressed genes among all samples, presented from outer circle to inner circle, COL.Blue, COL.Dark, COL.Red, COL.White, MES.Blue, MES.Dark, MES.Red, and MES.White, respectively **(C)**. Cluster analysis of DEGs based on gene expression of all samples **(D)**. Venn diagrams showing the number of DEGs among 16 comparisons **(E)**.

To investigate the effects of diffuse light signaling on gene expression dynamics in MES and COL plasticity elongation during maize germination, we performed RNA-Seq analysis on the MES and COL of Zheng58 seedlings grew under red, blue, white, and dark conditions. Each sample, namely COL.Blue (COL in blue light), COL.Dark (COL in darkness), COL.Red (COL in red light), COL.White (COL in white light), MES.Blue (MES in blue light), MES.Dark (MES in darkness), MES.Red (MES in red light), and MES.White (MES in white light), had three biological replicates. A total of 184.03 G clean data was obtained from the analysis, with each sample consisting of 6.48-9.37 G; the Q30 value exceeded 91%; and the GC content distribution was 53.06-55.99% (**Supplementary Table 2**). After filtering low-quality reads, 79.98-90.80% were mapped to the Zea_mays B73_V4 reference genome (**Supplementary Table 2**). Multivariate analysis, PCA, of the RNA-Seq datasets showed that the transcriptomes from the four light environments were clearly separated into two groups along the tissue types, and all replicates were closely clustered (**Figure 3A**). The results demonstrated the tissue-specific of different genes that were expressed differentially in maize MES and COL in response to different light quality. These expression patterns might be the result of the differentiation and plasticity elongation of MES and COL in maize. As the sampling, sequencing, and gene quantification in this study were of good quality, and we were able to further identify different light-induced DEGs in maize MES and COL tissues.

### DEGs analysis and functional annotations

FPKM values were used to evaluate all gene expressions (**Figure 3B**); in total, 38,669 expressed genes were identified, including 4,667 novel genes (**Figure 3C**). We then compared the transcript abundance in different samples, and the DEGs in each comparison were 43 (COL.Red_v_COL.Blue)~9,399 (MES.Blue_v_MES.White) (**Figures 3D, E**). The findings suggested that the gene expressions in MES and COL of Zheng58 seedlings were different from those induced by diverse light stimulations, thus they may be more sensitive to light stimulations. For example, by comparing the 9,414/6,611 unique DEGs of COL/MES under red, blue, and white light conditions relative to darkness treatment, 639/44 common DEGs were identified in the three comparisons (**Figure 3E**); by comparing the 7,970/11,296 unique DEGs of COL/MES under red and blue light conditions relative to white light condition, 2,215/6,586 common DEGs were identified in the two comparisons (**Figure 3E**). In addition, 11,095 unique DEGs were identified in two tissue types of Zheng58 seedlings, and only 352 common DEGs were identified in MES_vs_COL under red, blue, white light and darkness stimulations (**Figure 3E**). The finding confirmed that a large number of DEGs expression may have tissue-specificity.

To gain additional insight into the potential mechanisms underlying plasticity elongation of MES and COL during maize germination in four light inductions, DEGs from all 16 comparisons were functionally classified using GO enrichment analysis. The most important terms were represented by “cell”, “cell part”, and “organelle” under cellular component, “binding”, “catalytic activity”, and “transcription regulator activity” under molecular function, as well as “cellular process”, “metabolic process”, and “response to stimulus” under biological process (**Supplementary Table 3**). Further, KEGG pathway analysis among 16 comparisons showed that “circadian rhythm-plant (map04712)”, “plant hormone signal transduction (map04075)”, “phenylpropanoid biosynthesis (map00940)”, and “starch and sucrose metabolism (map00500)” (**Supplementary Figure 1**) were critical processes in plasticity elongation of MES and COL during maize germination under different light conditions. Thereby, these findings enriched our knowledge of the key biochemical pathways and genes regulating plasticity elongation of MES and COL during maize germination through environmental stimulations, especially light condition, which may help to develop environment resilient elite maize varieties. Further studies of the DEGs involved in the above pathways are necessary.

### DEGs involved in circadian rhythm

**Figure 4:**
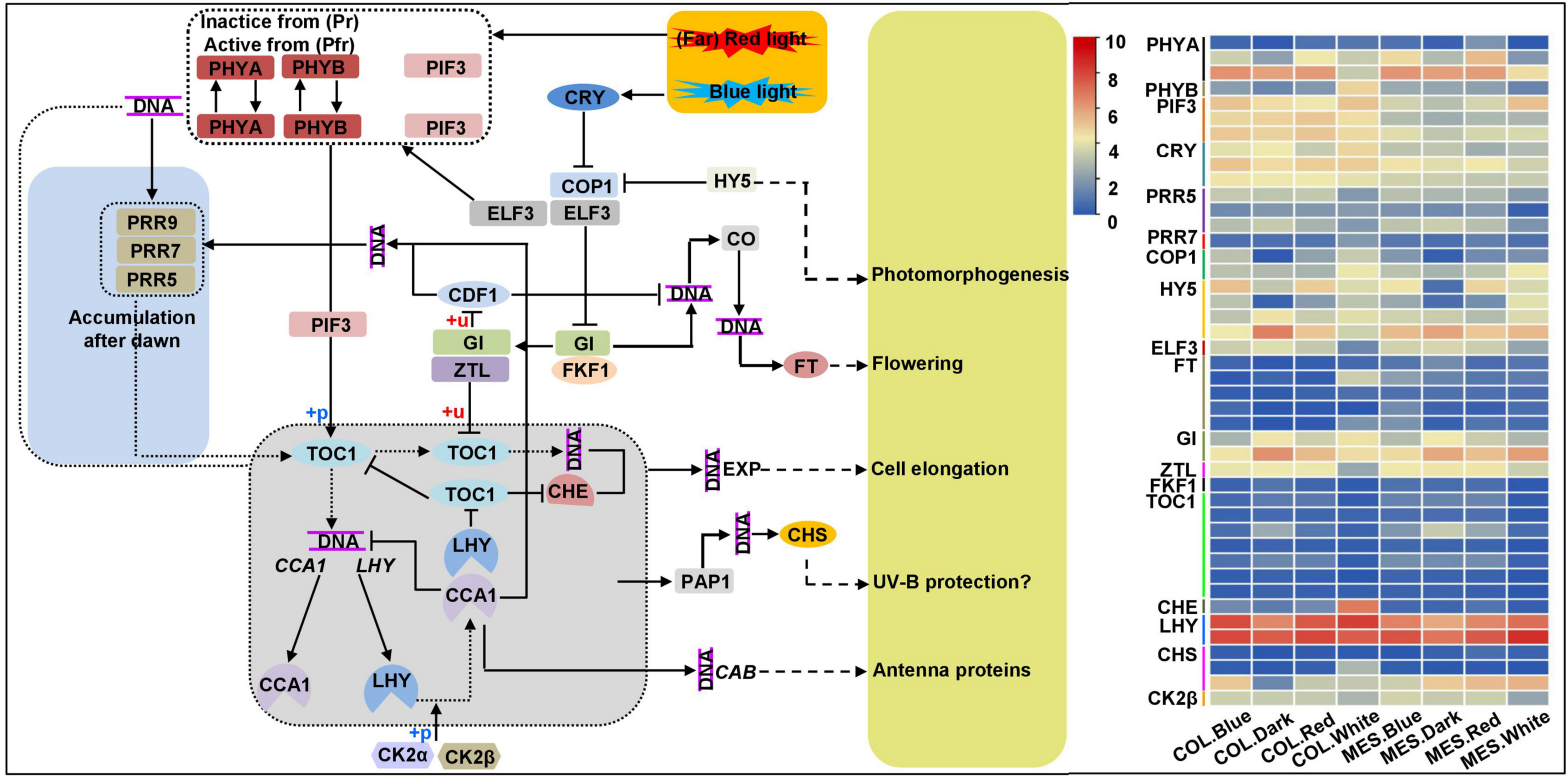
Circadian rhythm pathway and expression profiles of differentially expressed genes (DEGs) involved in circadian rhythm in mesocotyl (MES) and coleoptile (COL) of Zheng58 seedlings in four light stimulations.

To understand molecular mechanisms involved in the processes of plasticity elongation of MES and COL during maize germination in response to various light treatments, we identified the DEGs involved in circadian rhythm. Circadian rhythm is a well-known endogenous timekeeping system that can integrate various cues to regulate plant physiological functions for adapting to the changing environment and thus ensure optimal plant growth and development (Su et al., 2021). For example, elongated hypocotyl 5 (HY5) transcription factor (TF) mediates blue light signaling to *Arabidopsis* circadian clock and regulates its hypocotyl elongation (Anita et al., 2018). We identified 44 unique DEGs associated with circadian rhythm among 13 comparisons (**Figure 4**; **Supplementary Table 4**). These results revealed that the circadian clock could help maize to monitor light quality changes, resulting in changes in light-modulated gene expression. Subsequently, multiple DEGs related to the input pathway, central oscillator, and output pathway of the circadian clock are activated in MES and COL that might be involved in plasticity elongation, and plant hormone signaling pathways (Liu et al., 2022; Shi et al., 2022), sugar metabolism (Venkat and Muneer, 2022), and lignin synthesis (Harding et al., 2018) in MES and COL under different light controls.

### DEGs associated with plant hormone signal transduction

**Figure 5:**
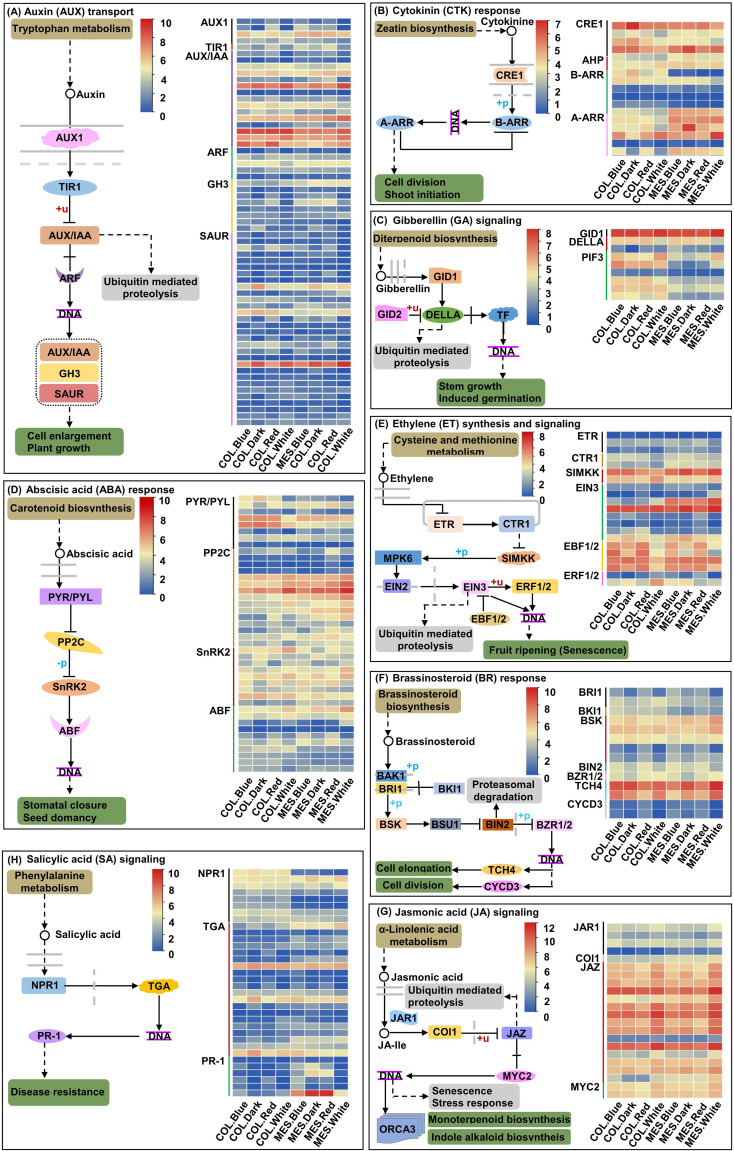
Eight plant hormones signal transduction pathways and expression profiles of differentially expressed genes (DEGs) involved in auxin (AUX; **A**), cytokinin (CTK; **B**), gibberellin (GA; **C**), abscisic acid (ABA; **D**), ethylene (ETH; **E**); brassinosteroid (BR; **F**), jasmonic acid (JA; **G**), and salicylic acid (SA; **H**) signaling pathway in mesocotyl (MES) and coleoptile (COL) of Zheng58 seedlings in four light stimulations.

Dark growth of MES, COL, or hypocotyl in the soil mainly depends on various phytohormones (Yue et al., 2021; Zhao et al., 2021a; Zhao et al., 2021b); on the soil surface, light activates trans-factors and initiate transition to photomorphogenic development (Kusnetsov et al., 2020). By exposing MES and COL of Zheng58 seedlings to red, blue, and white light environments and darkness, we identified 63 unique DEGs related to the AUX signaling pathway among 14 comparisons (**Figure 5A**; **Supplementary Table 5**); 19 unique DEGs associated with the cytokinin (CTK) signaling pathway among 11 comparisons (**Figure 5B**; **Supplementary Table 6**); nine unique DEGs associated with the GA signaling pathway among nine comparisons (**Figure 5C**; **Supplementary Table 7**); 42 unique DEGs regulating the ABA signaling pathway among 12 comparisons (**Figure 5D**; **Supplementary Table 8**); 21 unique DEGs controlling the ethylene (ETH) signaling pathway among 12 comparisons (**Figure 5E**; **Supplementary Table 9**); 14 unique DEGs for the BR signaling pathway among eight comparisons (**Figure 5F**; **Supplementary Table 10**); 22 unique DEGs associated with the jasmonic acid (JA) signaling pathway among ten comparisons (**Figure 5G**; **Supplementary Table 11**); and 34 unique DEGs related to the salicylic acid (SA) signaling pathway among 11 comparisons (**Figure 5H**; **Supplementary Table 12**).

### DEGs controlling cytoskeleton and cell wall organizations

**Figure 6:**
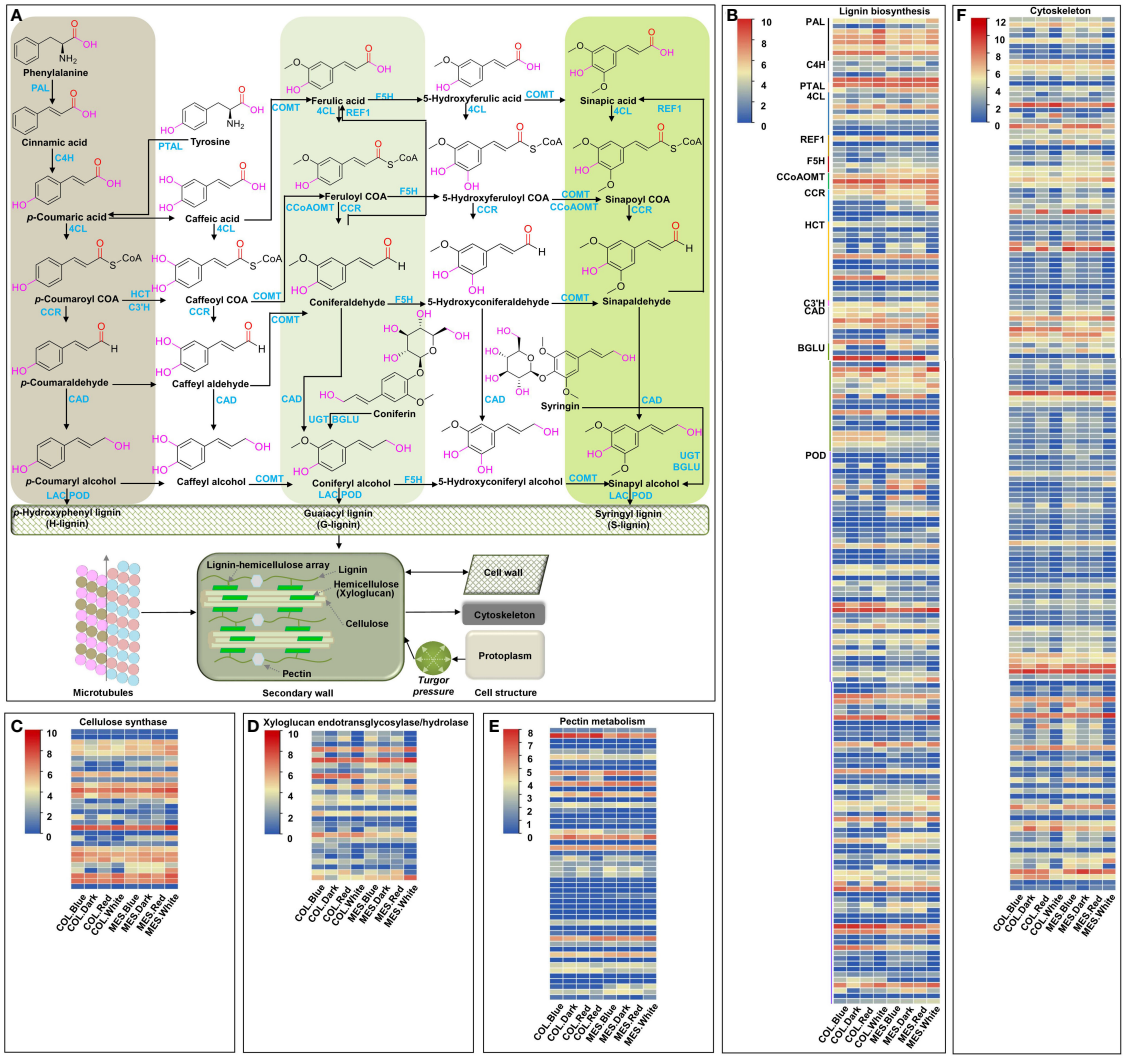
Cell wall organization and cytoskeleton, and expression profiles of differentially expressed genes (DEGs) involved in cell wall organization and cytoskeleton in mesocotyl (MES) and coleoptile (COL) of Zheng58 seedlings in four light stimulations. Diagram of cell wall organization and cytoskeleton, and lignin biosynthesis pathway **(A)**. Expression profiles of differentially expressed genes (DEGs) involved in lignin biosynthesis **(B)**, cellulose biosynthesis **(C)**, xyloglucan endotransglycosylase/hydrolase **(D)**, pectin metabolism **(E)**, and Cytoskeleton **(F)**.

Microtubules, one of the components of plant cytoskeleton, play an important role in many physiological activities, including maintaining cell morphology, controlling cell polar growth, and hypocotyl cell elongation (Li et al., 2011; Yue et al., 2021). We identified 163 unique DEGs controlling cytoskeleton among 13 comparisons (**Figure 6F**). The molecular function of GO annotation showed that these DEGs have structural constituents of the cytoskeleton (GO:0005200), microtubule motor activity (GO:0003777), ATPase activity (GO:0016887), peptidase activity (GO:0008233), ATP-dependent microtubule motor activity, plus-end-directed (GO:0008574), ATP binding (GO:0005524), GTP binding (GO:0005525), beta-tubulin binding (GO:0048487), and gamma-tubulin binding (GO:0043015) (**Supplementary Table 13**). The results indicated that these DEGs might affect the plasticity elongation of MES and COL in maize by regulating their microtubule-based movement (GO:0007018), microtubule severing (GO:0051013), and anisotropic cell growth (GO:0051211) under different light treatments (**Supplementary Table 13**).

Plant cell wall consists mainly of lignin, hemicellulose (xyloglucan), cellulose, and pectin; these cell wall organizations affect cell rigidity and relaxation, and determine cell elongation of maize MES under deep-seeding stress and exogenous EBR supply (Zhao et al., 2022a). In this study, 30 unique DEGs encoding cellulose synthase were identified in 11 comparisons (**Figure 6C**); GO annotation showed that these DEGs were involved in various biological processes including plant-type primary cell wall biogenesis (GO:0009833), cell wall organization (GO:0071555), and cellulose biosynthetic process (GO:0030244) (**Supplementary Table 14**). 28 unique DEGs associated with xyloglucan endotransglucosylase/hydrolase (XET/XTH, belonging to a glycosyl hydrolase family), were identified in 12 comparisons (**Figure 6D**). They displayed xyloglucosyl transferase activity (GO:0016762) and hydrolase activity, hydrolyzing O-glycosyl compounds (GO:0004553), resulting in involvement in xyloglucan metabolic process (GO:0010411), cell wall biogenesis (GO:0042546), and cell wall organization (GO:0071555) (**Supplementary Table 15**). 51 unique DEGs related to GDSL/SGNH-like Acyl-Esterase family, pectinesterase, plant invertase/pectin methylesterase inhibitor, glycosyl hydrolase family, pectate lyase, and pectin methyltransferase were identified in four comparisons (**Figure 6E**; **Supplementary Table 16**). Additionally, 184 unique DEGs involving in lignin biosynthesis were identified in 12 comparisons (**Figures 6A, B**; **Supplementary Table 17**). The above results indicated that red, blue, and white light up- or down-regulated the expression of a large number of genes regulating cell wall organization and cytoskeleton in maize MES and COL, resulting in cell wall remodeling, lignification, changes in movement of microtubules, and inhibition of their plasticity elongation during maize germination. At the same time, light-induced changes interconnected phytohormones signaling (Takahashi et al., 2012), sugar metabolism (Soga-Morimoto et al., 2021), and circadian rhythm (Harding et al., 2018) to form a complex regulatory network.

### Gene co-expression analysis both MES and COL in four light stimulations by WGCNA

**Figure 7:**
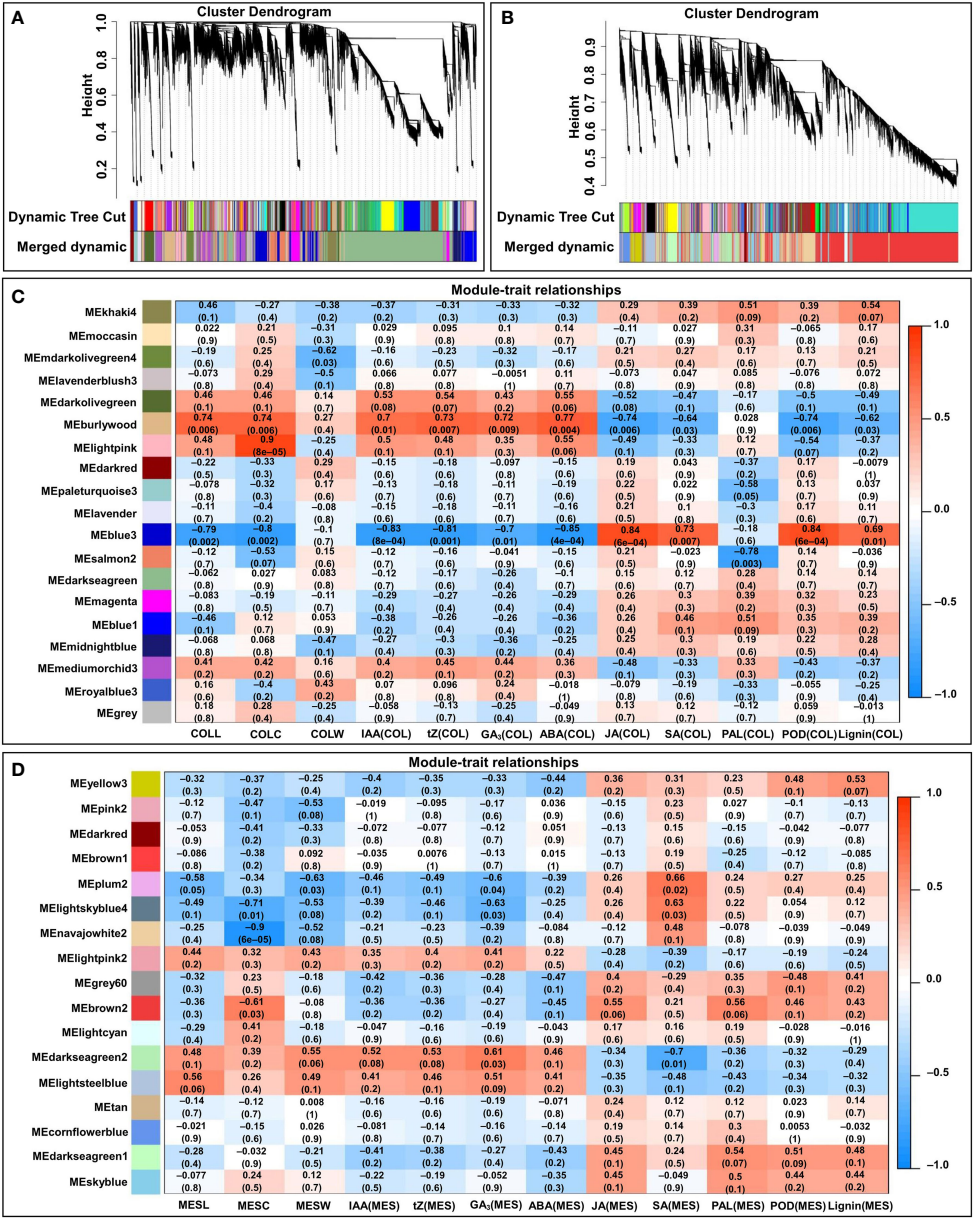
WGCNA of the transcripts in mesocotyl (MES) and coleoptile (COL) of Zheng58 seedlings in four light stimulations. Gene dendrograms from COL **(A)** and MES **(B)** were constructed using average linkage hierarchical clustering, each line represented one gene. The module color underneath the cluster tree showed the result of module assignment by the Dynamic Tree Cut. Correlations between modules eigengenes and COL tissue/traits **(C)**, and MES tissue/trait **(D)**. The color of each module was the same as that in **(A, B)**, respectively. The correlation coefficient (*r*) and *p*-value were shown in each cell. The traits including mesocotyl length (MESL), coleoptile length (COLL), mesocotyl coarse (MESC), coleoptile coarse (COLC), mesocotyl fresh weight (MESW), coleoptile fresh weight (COLW), indole-3-acetic acid content in MES/COL [IAA(MES/COL)], trans-zeatin content in MES/COL [tZ(MES/COL)], gibberellin 3 content in MES/COL [GA_3_(MES/COL)], abscisic acid content in MES/COL [ABA(MES/COL)], jasmonic acid content in MES/COL [JA(MES/COL)], and salicylic acid content in MES/COL [SA(MES/COL)], phenylalanine ammonia-lyase activity in MES/COL [PAL(MES/COL)], peroxidase activity in MES/COL [POD(MES/COL)], and lignin content in MES/COL [Lignin(MES/COL)].

To facilitate our understanding of the regulatory network of the tissue-specific and light quality induction-specific responses to plasticity elongation in maize MES and COL, expression data sets (FPKM > 1) from 24 MES and COL samples were subjected to WGCNA for finding clusters of gene sets with similar expression patterns (modules). A total of 17 and 19 co-expression modules (mergeCutHeight = 0.40) were identified in MES and COL tissues, respectively (**Figures 7A, B**). We then explored the correlations between the clusters (modules) using eigengene module (**Figures 7C, D**). Since COLL in four light treatments was positively correlated with IAA(COL) (*r*=0.939), tZ(COL) (*r*=0.906), GA_3_(COL) (*r*=0.867), and ABA(COL) (*r*=0.938), respectively (**Figure 2A**), we therefore focused on the burlywood module, which was significantly and positively correlated with COLL (*r*=0.74, *p*=0.006), IAA(COL) (*r*=0.7, *p*=0.01), tZ(COL) (*r*=0.73, *p*=0.007), GA_3_(COL) (*r*=0.72, *p*=0.009), and ABA(COL) (*r*=0.77, *p*=0.004), respectively (**Figure 7C**). As MESL in all light environments were positively correlated with IAA(MES) (*r*=0.928), tZ(MES) (*r*=0.964), GA_3_(MES) (*r*=0.959), and ABA(MES) (*r*=0.875), respectively (**Figure 2A**), we focused on darkseagreen2 and lightsteelblue modules that showed high and positive correlation to MESL (*r*=0.48 and 0.56, *p*=0.1 and 0.06), IAA(MES) (*r*=0.52 and 0.41, *p*=0.08 and 0.2), tZ(MES) (*r*=0.53 and 0.46, *p*=0.08 and 0.1), GA_3_(MES) (*r*=0.61 and 0.51, *p*=0.03 and 0.09), and ABA(MES) (*r*=0.46 and 0.41, *p*=0.1 and 0.2), respectively (**Figure 7D**).

**Figure 8:**
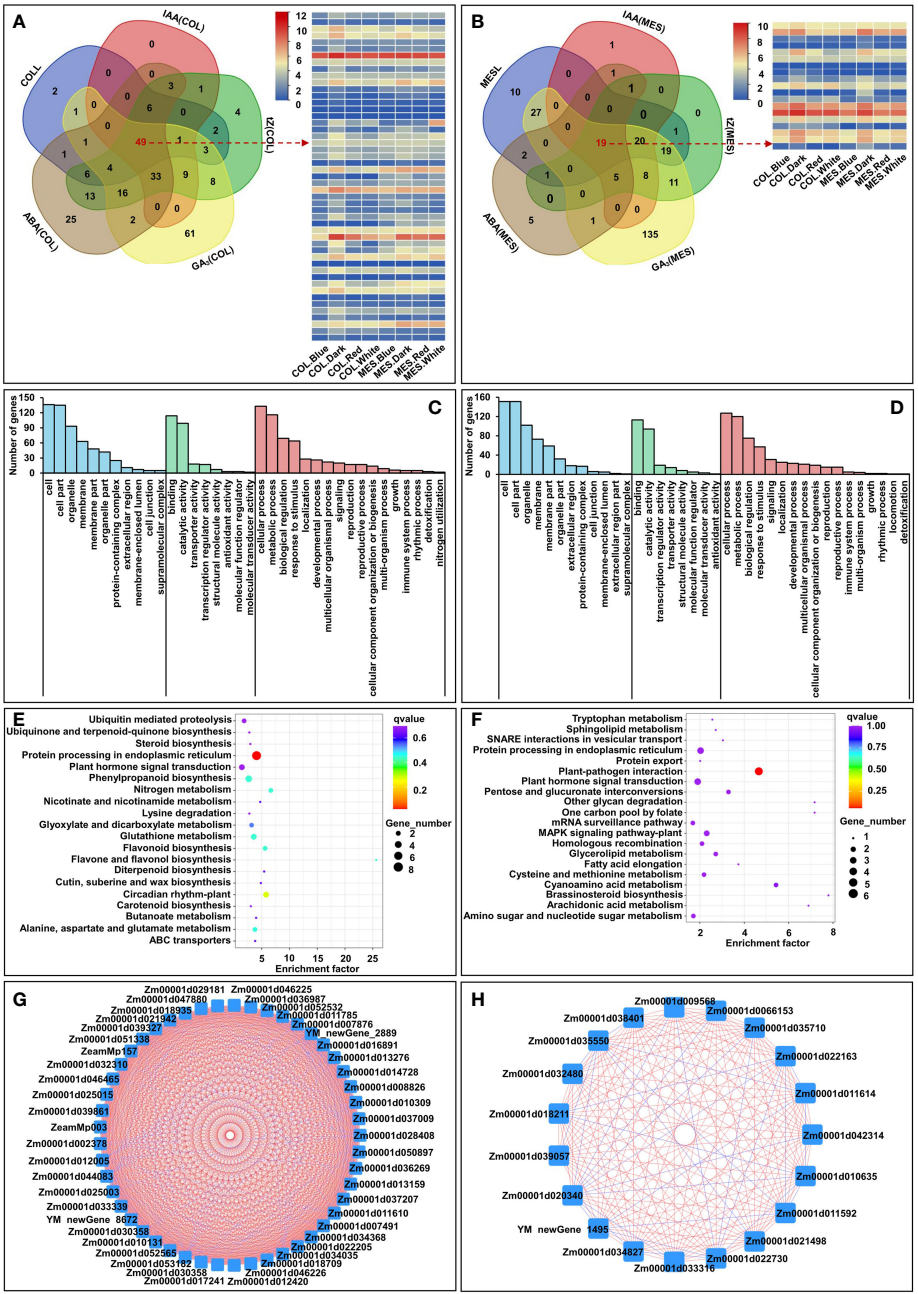
Venn diagram of burlywood module’ eigengenes in coleoptile (COL) to detect 251 unique genes and 49 common genes, which had a strong correlation with five corresponding traits, including coleoptile length (COLL), indole-3-acetic acid content in COL [IAA(COL)], trans-zeatin content in COL [tZ(COL)], gibberellin 3 content in COL [GA_3_(COL)], abscisic acid content in COL [ABA(COL)] [gene significance (GS) > 0.8], and the expression profiles of 49 common genes in burlywood module **(A)**. GO and KEGG analysis of 251 unique genes in burlywood module **(C, E)**. Hub genes network interaction in burlywood module in COL **(G)**. Venn diagram of darkseagreen2 and lightsteelblue modules’ eigengenes in mesocotyl (MES) to detect 267 unique genesand 19 common genes, which had a strong correlation with five corresponding traits, including mesocotyl length (MESL), IAA content in COL [IAA(MES)], tZ content in MES [tZ(MES)], GA_3_ content in MES [GA_3_(MES)] (GS > 0.7), ABA content in MES [ABA(MES)], and the expression profiles of 19 common genes in darkseagreen2 and lightsteelblue modules **(B)**. GO and KEGG analysis of 267 unique genes in darkseagreen2 and lightsteelblue modules (**D, F**). Hub genes network interaction in darkseagreen2 and lightsteelblue modules in MES **(H)**.

Further, we selected the burlywood module’s eigengenes, which had a strong correlation between gene expressions and the traits in COLL, IAA(COL), tZ(COL), GA_3_(COL), and ABA(COL) [gene significance (GS)>0.8]. The analysis identified 251 unique genes, and we then performed GO and KEGG analysis for those genes in a burlywood module to understand the biological functions (**Figure 8A**). GO biological processes were enriched in “cellular process”, “metabolic process”, and “biological regulation” (**Figure 8C**). Predominant pathways in KEGG were “Protein processing in endoplasmic reticulum”, “Circadian rhythm”, “Plant hormone signal transduction”, and “Phenylpropanoid biosynthesis” (**Figure 8E**). Similarly, we selected the darkseagreen2 and lightsteelblue modules’ eigenstates that had a strong correlation between gene expressions and the traits associated with MESL, IAA(MES), tZ(MES), GA_3_(MES), and ABA(MES) (GS>0.7). This analysis identified 267 unique genes (**Figure 8B**). GO analysis of these genes showed the predominance of “cellular process”, “metabolic process”, and “biological regulation” (**Figure 8D**); KEGG analysis revealed that majority of those genes belonged to “Plant-pathogen interaction”, “Plant hormone signal transduction”, and “Brassinosteroid biosynthesis” (**Figure 8F**). We therefore speculated that these pathways could play critical roles in plasticity elongation of MES and COL during different light exposure.

### Identification of hub genes and interaction network in modules

Hub genes were identified by setting module membership (MM)>0.5 and GS>0.8 were used to identify hub genes (Song et al., 2019). 49 and 19 common genes from COL and MES, respectively (burlywood module in COL, darkseagreen2 and lightsteelblue modules in MES) met the criteria as hub genes (**Figures 8A, B**), and were closely related to each other (**Figures 8G, H**). Within the burlywood module in COL, the hub genes, such as ARR1 protein-like isoform X1 (*Zm00001d011785*), two-component response regulator-like PRR1 (*Zm00001d017241*), and gigantea1 (*Zm00001d008826*) were involved in circadian rhythm (GO:0007623), response to blue light (GO:0009637), response to far red light (GO:0010218), and cell differentiation (GO:0030154). There were also genes, such as putative auxin efflux carrier PIN10a (*Zm00001d044083*), associated with auxin-activated signaling pathway (GO:0009734), regulated auxin polar transport (GO:0009926) and exhibited auxin efflux transmembrane transporter activity (GO:0010329). Gene encoding putative histidine kinase family protein (*Zm00001d012005*), this protein was involved in regulation of meristem development (GO:0048509), cellular response to abscisic acid stimulus (GO:0071215), cellular response to sucrose stimulus (GO:0071329). We also identified putative SnRK/SAPK family proteins (*Zm00001d018935*, *Zm00001d032310*, *Zm00001d03339*, and *Zm00001d039327*) that were associated with serine/threonine kinase activity (GO:0004674), and might involve in abscisic acid-activated signaling pathway (GO:0009738). The sterol 3-beta-glucosyltransferase (*Zm00001d025003*) showed brassicasterol glucosyltransferase activity (GO:0102203) and controlled sterol biosynthetic process (GO:0016126). The tubulin alpha-3 (*Zm00001d013159*), kinesin-like protein (*Zm00001d036987*), and myosin-12 isoform X1 (*Zm00001d013276*) had structural constituent of cytoskeleton (GO:0005200), microtubule motor activity (GO:0003777), and microtubule-based movement (GO:0007018). The E3 ubiquitin ligases (*Zm00001d052565*, *Zm00001d022205*, and *Zm00001d052565*) showed ubiquitin protein ligase activity (GO:1904264) and were responsible for endoplasmic reticulum unfolded protein response (GO:0030968). The glucose-6-phosphate 1-dehydrogenase 2 (*Zm00001d025015*), glutamate synthase 1 (*Zm00001d011610*), beta-1,3-galactosyltransferase 7 (*Zm00001d016891*), putative polyol transporter 1 (*Zm00001d021942*), and xylose isomerase (*Zm00001d039861*) were involved in cell wall polysaccharides synthesis (Sharples and Fry, 2007) (**Supplementary Table 18**). In addition, within the darkseagreen2 and lightsteelblue modules in MES, the hub genes, such as putative actin family protein (*Zm00001d032480*) belonged to cytoskeleton (GO:0005856). The 1-aminocyclopropane-1-carboxylate oxidase 1Acc oxidase (*Zm00001d018211*) was involved in ethylene biosynthetic process (GO:0009693). The vegetative cell wall protein gp1 (*Zm00001d034827*) regulated cell division. The MYB4 (*Zm00001d011614*) TF participated in multicellular organism development (GO:0007275), response to salicylic acid (GO:0009751), and cell differentiation (GO:0030154). The cytochrome P450 (*Zm00001d020340*) associated with isoflavone 3’-hydroxylase activity (GO:0048000) to regulate lignin biosynthesis. The CBL-interacting protein kinase 7 (*Zm00001d035710*) had protein serine/threonine kinase activity (GO:0004674) and intracellular signal transduction (GO:0035556). The glutaredoxin-C9 (*Zm00001d009568*) displayed cell redox homeostasis (GO:0045454). The probable 1-acylglycerol-3-phosphate O-acyltransferase (*Zm00001d006153*) with 1-acylglycerol-3-phosphate O-acyltransferase activity (GO:0003841). The protein SPEAR1 isoform X1 (*Zm00001d011592*) showed negative regulation of DNA-templated transcription (GO:0045892) and leaf development (GO:0048366) (**Supplementary Table 19**).

### Gene expression validation by qRT-PCR

**Figure 9:**
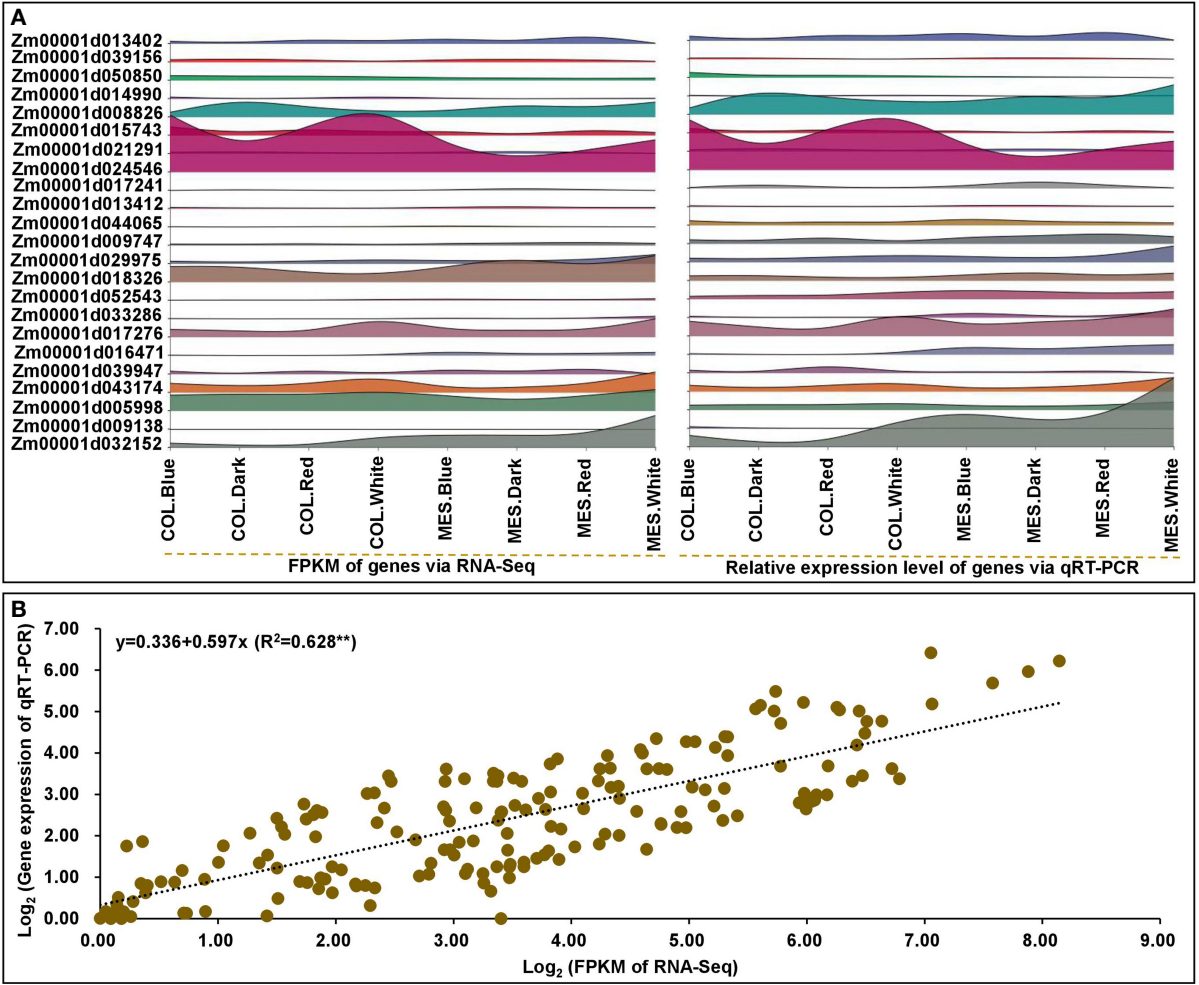
The quantitative real-time (qRT-PCR) expression analyses of 23 selected genes in mesocotyl (MES) and coleoptile (COL) of Zheng58 seedling in four light stimulations. Interactive layered area map for 23 selected genes comparisons by qRT-PCR and RNA-sequencing (RNA-Seq) were performed using Genescloud tool (https://www.genescloud.cn) **(A)**. Correlation between qRT-PCR and RNA-Seq data **(B)**.

To verify the reliability and validate RNA-Seq data, we analyzed the relative expression levels of 23 selected genes, including nine DEGs in circadian rhythm, six DEGs in phytohormone signaling, and eight DEGs in lignin biosynthesis, using qRT-PCR. The qRT-PCR expression patterns were in agreement with the relevant DEGs in RNA-Seq dataset (**Figure 9A**; **Supplementary Table 20**). There was a strong linear relationship between the RNA-Seq dataset and qRT-PCR expression levels of MES and COL in Zheng58 seedlings under four light treatments (y=0.336+0.597x; R^2 =^ 0.628^**^) (**Figure 9B**), indicating that the consistency between two analytical methods.

## Discussion

### Influence of light on plasticity elongation and physiology of maize MES and COL

Light, including varying intensity, spectral quality, and duration, is one of the most critical environmental factors that impact plant development. The inhibition of plasticity elongation of MES and COL by light is a well-characterized phenomenon in maize (Walton and Ray, 1981; Markelz et al., 2003). In general, the elongation of MES and COL in maize was immediately inhibited by light exposure and significantly promoted in darkness. MES elongation in 4-day-old etiolated maize seedlings could be dramatically inhibited by red, far-red, and blue light within less than three hours after the onset of irradiation (Schopfer et al., 2001). During the first three days of elongation period, light could decrease the growth rate of maize COL, and the influence of blue light was more substantial than red light among different spectra on the elongation of COL (Parvez et al., 1996). Consistent with prior studies, we observed that the MESL and COLL levels in Zheng58 seedlings decreased by 47.7, 30.5 and 61.7%, and 49.5, 43.0 and 37.9% after red, blue, and white light exposure for five days (**Figure 1B**). These experiments confirmed that different light quality have similar inhibition elongation trend on MES and COL in maize with the order of blue light > red light > white light > darkness (Zhao and Niu, 2022). Dark-grown maize seedlings exposed for one or more hours to white light and then returned to darkness developed MES with enlarged apical diameters and their swelling was the result of transverse cell enlargement, not an increased cell numbers (Camp and Wickliff, 1981). Our results showed that compared to darkness, five days of red, blue, and white light induced 10.7 and 6.4%, 13.7 and 14.0%, as well as 32.5 and 21.4% decreases in MESC and COLC of Zheng58 seedlings (**Figure 1B**). We speculated that the duration of the diffusion and variation in light treatments caused this difference in transverse growth of maize MES and COL cells in the same region. We also found that the MES was more sensitive to many of the same light stimuli than COL (**Figures 1A, B**), which may be related to the genetics of adaptive responses of these two tissue types to light signaling during their domestication. The quantitative dosage of red light (12 mM m^-2^ s^-1^) inhibits about 50% of MES growth in maize, while an influx of 1 mM m-^2^ s-^1^ decreases the growth about 20% of the dark control (Jones et al., 1991). In rice seedlings, the MES shows little growth in darkness for two more days after a 1 min pulse of white light at 10 mM m^-2^ s^-1^ (Feng et al., 2017). Consequently, the negative regulation of light on the plasticity elongation of both tissues in maize resulted from a comprehensive effect of spectral quality, intensity, and duration of light exposure. This finding was surprising given the shorter MES detected in the tropical germplasms compared with the U.S./Canadian corn belt lines, indicating that a loss of light responsiveness at the seedling emergence stage had accompanied selection by breeders in northern temperature regions (Markelz et al., 2003). Light response pathways or photo-morphogenesis may be an ideal target for maize improvement in the future.

A very efficient cross-talk has evolved between light signaling and endogenous phytohormones in plants, which had been frequently invoked as effectors of light responses (Riemann et al., 2003; Feng et al., 2017). The phytohormones profiling in the elongating MES of rice in response to light exposure over time has revealed that light could inhibit MES elongation by increasing JA level with concomitant decrease in IAA, GA_3_, and tZ accumulation (Feng et al., 2017). When three-day-old etiolated maize seedlings were treated with red light, the photo-signal was recognized by the PHY of MES intercalary meristem, which subsequently hampered IAA transport from COL into MES resulting in drastic reduction of MES growth (Polevoi, 2001). AUX hypothesis proposed that red light inhibited MES growth of maize mainly by reducing the IAA supply from COL (Lino, 1982). Quantitative proteomic and genomic studies demonstrated that light-induced inhibition of elongation of MES in maize was not only depended on AUX reduction but also on BR (Kutschera and Wang, 2016). JA was rapidly stimulated by a factor of 10 to 20 in rice COL in response to red light exposure (Feng et al., 2017). This study also showed that compared with darkness control, red, blue, and white light exposure caused significant decrease level of IAA, tZ, GA_3_, and ABA in MES and COL of Zheng58 seedlings, while substantially increased JA and SA contents (**Figure 1C**). It is likely that these endogenous phytohormones maintain a subtly dynamic balance during MES and COL plasticity elongation in response to different light stimulations. Furthermore, Pearson pairwise correlation analysis showed that there was extremely close correlation among six phytohormones level in MES and COL and plasticity elongation of MES and COL under four light treatments (**Figure 2A, B**). Therefore, polar transport or interactions of phytohormones exist between MES and COL in maize. As various phytohormones were the central regulator of plasticity elongation of both tissues in maize under different spectral quality of light, their dynamic changes are likely to be controlled by up- or down-regulations of corresponding phytohormones-related genes.

PAL is the first key enzyme in lignin biosynthesis (Zhao et al., 2022a), the inhibition of PAL activity could enhance lignin deposition in the cell wall followed by a reduction of maize root growth (Siqueira-Soares et al., 2013). Continuous white or blue light induced both anthocyanin synthesis and enhanced PAL activity in MES of maize seedlings, but short red light had no impact on PAL activity (Duke and Naylor, 1976). However, exposure to red light increased PAL activity in leaves of 26-28-day-old *Arabidopsis* wild type and mutant *hy3* (deficient in PHYB) plants (Shmarev et al., 2020). Consistently, exposure to red, blue, and white light enhanced PAL activity in MES and COL of Zheng58 seedlings in our study (**Figure 1C**). Red-light-elicited increase of polyamine oxidase (PAO) activity was correlated with the growth inhibition of the outer tissues in the apical, growing zone of maize MES because of its role in increasing H_2_O_2_ accumulation (Laurenzi, 1999). Increased H_2_O_2_ accumulation subsequently enhanced POD activity (Zhao et al., 2021b), which then oxidized p-coumaryl-/coniferyl-/sinapyl-alcohol on the cell wall to polymerize into lignin monomers (Zhao et al., 2022a) resulting in the modulation of growth, cell wall stiffening, and differentiation in maize MES (Laurenzi, 1999). In our study, POD activity in MES and COL of Zheng58 was increased by 130.1~603.6% along with 50.4~185.8% increase in lignin accumulation in response to red, blue, and white light (**Figure 1C**) that were consistent with previous studies. However, this phenomenon of formation of lignin in maize MES and COL was reversed under deep-seeding stress (Zhao and Zhong, 2021). Therefore, breeding for low lignin accumulation in both MES and COL of maize could contribute to the development of elite maize varieties with longer MES and/or COL to cope with adverse environments.

In parallel, cellular osmotic properties are among the important factors that regulate the rate of cell expansion (Chen et al., 2016). It is assumed that the amount of osmotic solutes, including sugar, in cells determines the amount of water uptake, thus controlling the rate of cell growth. Soga-Morimoto et al. (2021) reported that sugar accumulation in maize MES and COL cells was reduced with subsequent suppression of cell growth when etiolated seedlings were subjected to white light irradiation. Kutschera and Niklas (2013) demonstrated that the hypocotyl growth of *Helianthus annuus* L. was associated with turgor-driven enlargement of cells in darkness and white light treatments, and the turgor maintenance during hypocotyl elongation was caused by sucrose catabolism, resulting in the generation of osmoregulant (such as monosaccharide). In summary, sucrose is the main export form of sugar, the translocation process of sugar from the storage tissues to MES, COL, or hypocotyl may be suppressed by light. Different light-mediated metabolism of starch and sucrose in maize MES and COL warrants further study.

### Potential molecular mechanisms and DEGs analysis of maize plasticity elongation of MES and COL in light stimulations

The MES or COL of rice (Riemann et al., 2003; Feng et al., 2017) and hypocotyl of *Arabidopsis* (Anita et al., 2018; Lan et al., 2023) had been widely used as experimental system to reveal the molecular mechanisms underlying the light-repression of plant growth. So far, there has been very limited progress in studying the molecular mechanism of maize plasticity elongation of MES and COL under various light spectra. In this study, we used Illumina RNA-Seq to analyze the transcriptomic changes in MES and COL tissues of Zheng58 seedlings across red, blue, and white light irradiation and darkness. Overall, number of identified DEGs ranged from 43 (COL.Red_v_COL.Blue) to 9,399 (MES.Blue_v_MES.White) among 16 comparisons (**Figure 3E**). It was astonishing that compared to MES or COL at darkness, only 44 common DEGs were identified in MES at red, blue, and white light irradiation, which was much less than 639 common DEGs identified in COL at above three light treatments. However, compared to white light, 6,586 and 2,215 common DEGs were detected under red and blue light in both tissue types. Additionally, only 352 common DEGs were found among the four comparisons in two tissue types treated with same four light conditions (**Figure 3E**). So, light exposure could induce the changes in expression patterns of a large number of genes in MES and COL, and the patterns could vary greatly with tissue types and light quality. Based on GO and KEGG analysis, we then focused on several important pathways and corresponding DEGs.

To sense and respond to daily changes imposed by Earth’s rotation, organisms of all life form have evolved an endogenous 24-h timer with daily rhythmic functions (circadian clock). Previously, Nusinow et al. (2011) and Li et al. (2020) reported that under normal photoperiodic treatments, the circadian regulation of *Arabidopsis* growth was standard, which could be predicted from robust hypocotyl elongation. PHYs and CRYs are important plant photoreceptors. PHY inhibited hypocotyl and MES elongation under light stimulations (Yang et al., 2016) possibly through the interactions of PHYB with two AUX response factors (ARF7 and ARF9) and an AUX/IAA protein-responsive protein IAA14 that inhibited their transcriptional activities (Li et al., 2021). CRY1 N- terminus found to be involved in CRY1 signaling and implicated in the inhibition of GA, BR, AUX-responsive gene expression that negatively impact hypocotyl elongation (Wang et al., 2016). In this study, we detected three PHYA (*Zm00001d008542*, *Zm00001d013402*, and *Zm00001d033799*), a PHYB (*Zm00001d047632*), and three CRY (*Zm00001d003477*, *Zm00001d01915*, and *Zm00001d050850*) DEGs showed varied expression levels in multiple comparisons (**Figure 4**; **Supplementary Table 4**). Thus above DEGs sensed light-mediated circadian rhythms and involved in multiple phytohormones signaling in MES and COL. HY5 (a bZIP TF) was a signaling hub acting downstream of several photoreceptors and a key mediator of photo-morphogenesis, which interacted with COP1 (E3 ubiquitin-protein ligase RFWD2; a negative regulator of photo-morphogenesis) (Anita et al., 2018). The HY5-COP1 module acted a common signaling node that mediated cross-talk among multiple pathways, thereby, enhancing the plant phenotypic plasticity (Akankshaa et al., 2020). Our results also confirmed this conclusion, the expression patterns of identified four HY5 (*Zm00001d015743*, *Zm00001d046402*, *Zm00001d008734*, and *Zm00001d039658*) and two COP1 (*Zm00001d014990* and *Zm00001d052138*) DEGs were same in both tissues under four light exposures (**Figure 4**; **Supplementary Table 4**). Nusinow et al. (2011) and Li et al. (2020) identified an evening complex (EC)-composed of two early flowering proteins (ELF4 and ELF3) and LUX ARRHYTHMO; five PRRs gene family members (PRR3, PRR5, PRR7, PRR9, and TOC1), which could regulate the abundance and activity of PIF3 (phytochrome-interacting factor 3), a positive regulator of plant cell elongation (Martin et al., 2018). We found that ELF3 (*Zm00001d039156*) DEG was down-regulated in MES and COL under red/blue light_v_white light, but up-regulated under white light_v_darkness (**Figure 4**; **Supplementary Table 4**). Similarly, one of three PRR5 DEGs, *Zm00001d006212*, was down-regulated in MES and COL under red/blue light_v_white light, other two DEGs (*Zm00001d004875* and *Zm00001d021291*) were down-regulated in MES under red/blue light_v_white light (**Figure 4**; **Supplementary Table 4**). A PRR7 DEG (*Zm00001d007240*) was common in both COL.Red_v_COL.White and MES.White_v_COL.White comparisons (**Figure 4**; **Supplementary Table 4**). Most of the seven TOC1 DEGs were down-regulated in MES under red/blue light_v_white light, but they were up-regulated in MES and COL under white light_v_Darkness resulting in the inhibition of *Zm00001d024783* (PIF3) expression in both tissues under white light_v_darkness (**Figure 4**; **Supplementary Table 4**). These findings well explain the light-mediated inhibition of maize MES and COL elongation. Moreover, whether other DEGs involved in circadian rhythm (**Figure 4**) have regulatory roles in plasticity elongation of both tissues under different light regimes need to verify in the future.

Phytohormones play key roles in light-dependent regulation of MES and COL elongation that have frequently acted as effectors of light responses (Feng et al., 2017). For AUX signaling, we found that six GH3 (encoding IAA-amido synthetases, help to maintain AUX homeostasis by conjugating excess IAA to amino acids) and 14 AUX-responsive SAUR DEGs were down-regulated in MES and COL under red and blue light inductions (**Figure 5A**; **Supplementary Table 5**). Similarly, in rice MES, two GH3 and three SAUR genes were down-regulated in response to light at three time points (Feng et al., 2017). Previously, Zhao et al. (2013) reported that *OsGH3.1* mutant contained low level of free IAA and insensitive to IAA, which was consistent with decreased IAA content in both MES and COL exposed to different light spectra in our study (**Figure 1C**). Spartz et al. (2012) found that *AtSAUR24* could promote cell expansion and hypocotyl growth in *Arabidopsis*. It is possible that decreased IAA content in both MES and COL in maize under different light treatments might be linked to the down-regulation of GH3 and SAUR DEGs in our study. Lv et al. (2020) reported that non-canonical AUX/IAA protein IAA33 maintained root distal stem cell identity and negatively regulated AUX signaling by interacting with ARF10 and ARF16. Down-regulation of six of 15 AUX-responsive DEGs in two tissues by red and blue light stimulations might be related to similar mechanism (**Figure 5A**; **Supplementary Table 5**). HY5 is a point of convergence between CRY and CTK signaling, and CRY1 and CTK signaling could increase HY5 protein level (Vandenbussche et al., 2007). Meanwhile the level of tZ in MES and COL was significantly down-regulated by blue light exposure compared to other three treatments (**Figure 1C**). Perhaps the regulation of plant plasticity growth by CTK signaling was closely associated with circadian rhythm pathway. It was reported that CTK mediated hypocotyl and root growth is regulated through CTK receptor *Arabidopsis* histidine kinase 3 (AHK3) (Novak et al., 2015). Three CRE1 DEGs encoded AHK (*Zm00001d012005*, *Zm00001d014297*, and *Zm00001d042312*) were up-regulated in COL under red/blue/white light_v_darkness (**Figure 5B**; **Supplementary Table 6**), implying that CRE1 DEGs expression may have tissue-specificity. After GA binding, GID1 (a GA receptor) interacted with DELLA protein (a GA repressor), leading to DELLA polyubiquitination and degradation by the E3 ubiquitin ligase, and subsequent ease of transcriptional inhibition of DELLA on PIF and hypocotyl elongation promotion (Bai et al., 2012). Despite a GID1 DEG (*Zm00001d038165*) was up-regulated and two DELLA DEGs (*Zm00001d033680* and *Zm00001d044065*) were down-regulated in MES under red and blue light as well as six PIF3 DEGs were up-regulated in multiple comparisons (**Figure 5C**; **Supplementary Table 7**), GA signal transduction did not show positive impact on MES and COL elongation in our study. Besides, ABA could increase DELLA level to impair GA signaling and ultimately inhibit AUX biosynthesis genes expression (Lorrai et al., 2018). In this study, eight PYR/PYL (ABA receptor family) DEGs were varied negative expression levels (**Figure 5D**; **Supplementary Table 8**), which might resulted in low accumulation of ABA in MES and COL by different light exposure (**Figure 1C**).Ten PP2C and four SnRK2 DEGs were up-regulated in MES and COL by red and blue light exposure (**Figure 5D**; **Supplementary Table 8**) that might led to enhanced PP2C (protein phosphatase 2C) activity followed by activation of SnRK2 (serine/threonine-protein kinase SRK2).

Generally, ETH activated PIF3 *via* EIN3 (ethylene-insensitive protein 3) to promote growth as observed in *Cucumis sativus* hypocotyl (Dan et al., 2003). In contrasting scenario, ETH enhanced ERF1/2 (ethylene-responsive transcription factor 1) under darkness and inhibited the elongation of hypocotyl (Zhong et al., 2012). In this study, two EIN3 DEGs (*Zm00001d016924* and *Zm00001d050861*) were down-regulated in MES by red and blue light stimulations, however, two ERF1/2 DEGs (*Zm00001d034920* and *Zm00001d019734*) were up-regulated in COL under blue light (**Figure 5E**; **Supplementary Table 9**). In rice, *OsBSK3* (BR-signaling kinase, a downstream component in BR signaling) interacted with *OsGSK3* (a conserved GSK3-like kinase that regulates the phosphorylation of CYC U2) and inhibited dephosphorylation of *OsGSK3* (Tian et al., 2022). BR promoted rice MES elongation *via OsGSK2*, a conserved *GSK3*-like kinase controlling the phosphorylation of CYC U2 (Sun et al., 2018). We found that red and blue light enhanced the expression of three BSK DEGs (*Zm00001d027523*, *Zm00001d048345*, and *Zm00001d030021*) in MES (**Figure 5F**; **Supplementary Table 10**). BR controlled cell division by promoting the accumulation of cytoskeleton protein F-actin, and the expression of CDC48 (cell division cycle protein 48) and CYCD2 (cyclin D2) (Zhan et al., 2020). In our study, two CYCD3 DEGs (*Zm00001d019696* and *Zm00001d005293*) were down-regulated in MES and COL by red and blue light exposure, respectively (**Figure 5F**; **Supplementary Table 10**). Additionally, BR and GA interaction could promote DELLA degradation. DELLA inhibited BZR1-DNA binding both *in vitro* and *in vivo* by directly interacting with BZR1 (Brassinozale-Resistant 1), which subsequently activated BZR1 and regulated seedling etiolated growth (Bai et al., 2012). We identified one BZR1/2 DEG (*Zm00001d006677*) that was down-regulated in both MES.White_vs_MES.Dark and COL.White_vs_COL.Dark comparisons (**Figure 5F**; **Supplementary Table 10**), predicting that this gene could interact with multiple DELLA genes to co-regulate BR and GA signaling. We also identified two TCH4 (xyloglucosyl transferase) DEGs (*Zm00001d009309* and *Zm00001d002410*), downstream of BR signaling response, and were up-regulated in MES under red and blue light treatments (**Figure 5F**; **Supplementary Table 10**); these two genes might be involved in re-modelling of MES cell wall structures and inhibition of MES cell elongation under red and blue stimulations. JA-mediated inhibition of hypocotyl elongation was dependent on JA receptor COI1 (coronatine-insensitive protein 1) and signaling components, such as repressor proteins JAZs (jasmonate ZIM domain-containing proteins) and transcription activators MYC2/MYC3/MYC4 that activated the expression of HY5 to repress cell elongation-related genes including SAUR62 and EXP2 (expasion2) (Yi et al., 2020). We found that one COI (*Zm00001d047848*), 15 JAZ, and two MYC2 (*Zm00001d047017* and *Zm00001d030038*) DEGs were up-regulated in MES by red and blue light exposure (**Figure 5G**; **Supplementary Table 11**). Similarly, Feng et al. (2017) demonstrated that six JAZ genes were up-regulated in rice MES in response to white light exposure. These results indicate that JA signal transduction is involved in light-dependent inhibition of plants MES elongation.

In this study, we detected 163 DEGs encoding CLIP-associated protein, WVD2-like protein, microtubule-associated protein, katanin p60 ATPase-containing subunit A1, katanin p80 WD40 repeat-containing subunit B1 homolog, mitotic-spindle organizing protein, kinesin-like protein, WD-40 repeat family protein, tubulin beta chain, gamma-tubulin complex component 3, separase isoform X2, plasma membrane-associated cation-binding protein 1, and C-terminal binding protein AN among 13 comparisons (**Figure 6F**; **Supplementary Table 13**). Several groups have demonstrated the involvement of these proteins in plant developmental processes, including hypocotyl cell elongation. For example, BZR1 directly targeted and up-regulated MDP40 (microtubule destabilizing protein 40), which subsequently promoted *Arabidopsis* hypocotyl cell elongation (Wang et al., 2012). ETH signaling up-regulated MDP60 expression *via* PIF3 binding to the MDP60 promoter and modulated hypocotyl cell elongation by altering cortical microtubules in *Arabidopsis* (Ma et al., 2018). The microtubule-severing enzyme katanin (particularly p80 subunit KTN80 and p60 subunit KTN1) triggered dynamic reorientation of cortical microtubule arrays that were crucial for cell elongation, cell wall biosynthesis, and phytohormones signaling (Wang et al., 2017). Light mediated regulation of WAVE-DAMPENED 2-LIKE3 (WDL3), a microtubule regulatory protein of the WVD2/WDL family and was involved in *Arabidopsis* hypocotyl cell elongation, by an ubiquitin-26S proteasome-dependent pathway was also reported by Liu et al. (2013). Le et al. (2005) demonstrated that transverse and longitudinal patterns of microtubules were related with rates of elongation of *Arabidopsis* hypocotyl in darkness, however, only longitudinally orientated microtubules were associated with hypocotyl growth arrest under light stimulation. CLASP (CLIP-associated protein) promoted microtubules stability and involved in both cell division and cell expansion; its T-DNA insertion mutants were hypersensitive to microtubule-destabilizing drugs and exhibited more sparsely populated microtubules in roots and shorter plant stature (Ambrose et al., 2007). Hence these results might provide new insights into cytoskeleton establishment and plasticity elongation in both MES and COL in maize by different light exposures.

Cell wall organization is also an important factor affecting cell elongation and expansion. The primary cell walls of plants consist of cellulose microfibrils tethered mainly by xyloglucans and embedded in a highly hydrated pectin matrix. Cellulose synthases were necessary for normal cell elongation (Fagard et al., 2000), while XET/XTH (Van Sandt et al., 2007), expansin, and endoglucanase (Cosgrove, 2016) acted as cell wall-loosening enzymes. In this study, 30 cellulose synthase and 28 XET/XTH DEGs were identified and most of them were differentially down-regulated in multiple comparisons; expression of 14 DEGs for cellulose synthase were down-regulated under white light in MES, and blue light caused negative expression of 11 and 12 XET/XTH DEGs in MES and COL, respectively (**Figure 6C**, **7D**; **Supplementary Tables 14, 15**). White light mainly inhibited expression of cellulose synthase genes in MES, conversely, blue light mainly down-regulated XET/XTH genes in both tissues (**Figure 6C**, **7D**; **Supplementary Tables 14, 15**). Moreover, we detected 51 DEGs associated with pectin metabolism that might be involved in the regulation of pectin biosynthesis or cell wall modification (**Figure 6E**; **Supplementary Table 16**). Lignin is an important component of the secondary wall of plant cells, lignin accumulation was directly related to cell wall rigidity (Zhao et al., 2021b). In this study, seven and five DEGs associated with PAL were up-regulated in MES and COL by red and blue light exposure (**Figure 6B**; **Supplementary Table 17**); the expression patterns of PAL genes were consistent with PAL activity (**Figure 1C**). More than half of the DEGs (approximately 56.5%) regulating POD activity were identified in multiple comparisons (**Figure 6B**; **Supplementary Table 17**), and up- or down-regulation of these POD genes were likely to be related to H_2_O_2_ production in MES and COL exposed to different light condition. Consistently, DEGs for nicotinamide adenine dinucleotide phosphate oxidase (NADPH), which was a source of H_2_O_2_ production, were also varied expression in different comparisons. Multiple TFs, including MYB, NAC, WRKY, and LIM, were involved in lignin biosynthesis pathways (Zhao et al., 2022a). How these TFs interacted with genes involved in lignin biosynthesis, and the function of them to regulate plasticity elongation of maize MES and COL under different light treatments remains to be studied.

**Figure 10:**
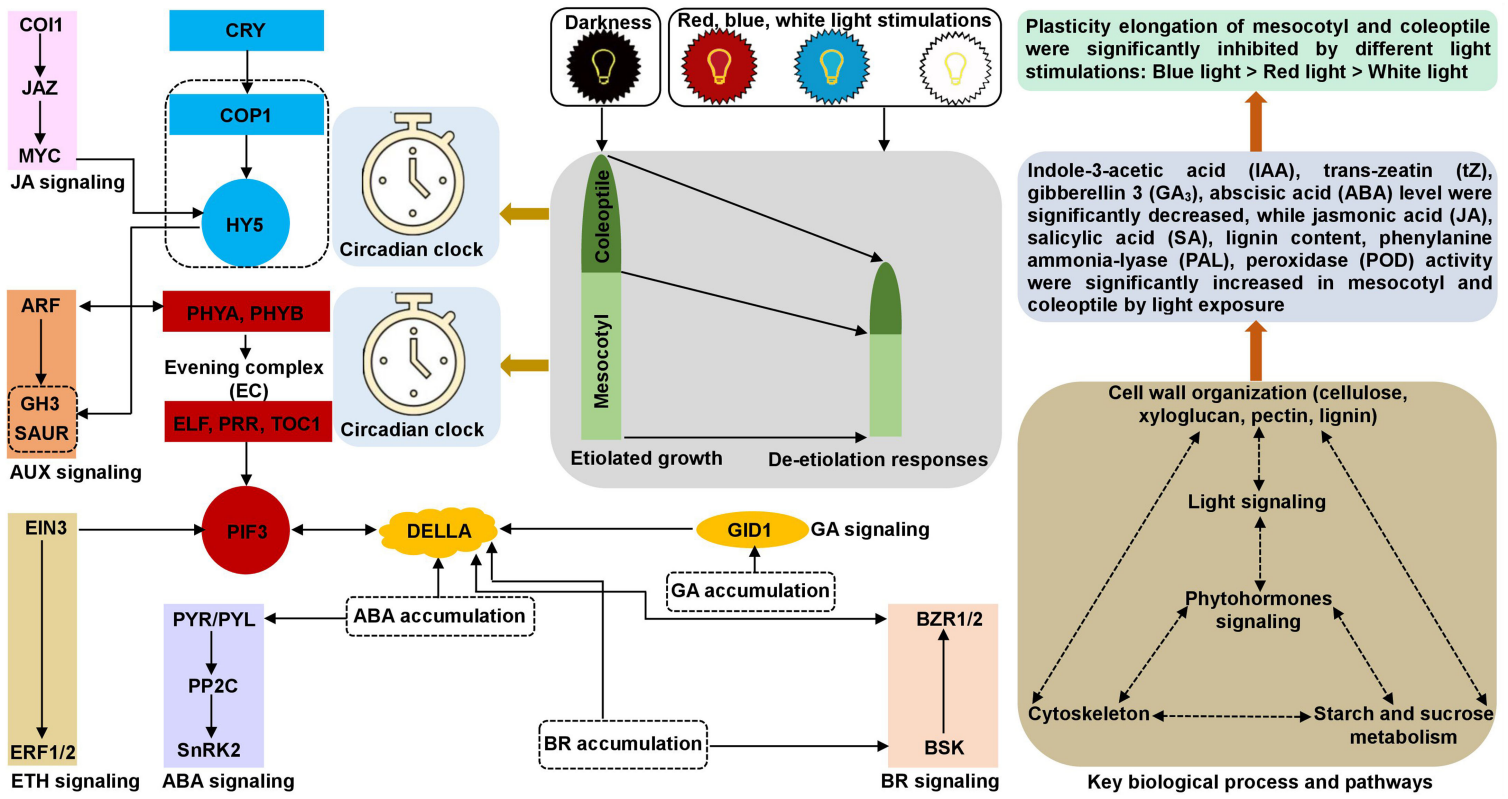
Molecular network underlying the maize plasticity elongation of mesocotyl and coleoptile in response to red, blue, and white light exposure and darkness. The synergy and antagonism of key genes/transcription factors in circadian rhythm, phytohormones signal transduction, cytoskeleton, cell wall organization, starch and sucrose metabolism processes activate/inhibit the expression of essential downstream genes and changes the accumulation of corresponding metabolites, resulted in different light inhibited plasticity elongation of maize mesocotyl and coleoptile.

In summary, we established a possible molecular network underlying the inhibition of maize plasticity elongation by MES and COL in red, blue, and white light stimulations (**Figure 10**). These findings not only provided a new perspective on the etiolated growth and de-etiolation process in response to different light exposure to maize, but also laid a theoretical foundation for further functional analysis of promising genes and/or select target(s) for gene editing and breeding applications to develop abiotic stress tolerant varieties.

## Data availability statement

The datasets presented in this study can be found in online repositories. The names of the repository/repositories and accession number(s) can be found in the article/**Supplementary Material**.

## Author contributions

XZ, YN, ZH, and BZ carried out the transcriptomics analysis and drafted the manuscript. XZ, XB, and TM participated in material collection and performed the statistical analysis. XZ and YN conceived of the study and participated in its design. All authors contributed to the article and approved the submitted version.
