# Supplementary material for: New insights into light spectral quality inhibits the plasticity elongation of maize mesocotyl and coleoptile during seed germination
Source: Front Plant Sci. 2023 Mar 15;14:1152399. doi: 10.3389/fpls.2023.1152399 (PMC10050570; doi:10.3389/fpls.2023.1152399)
Supplement: Supplementary file 2 [file DataSheet_1.pdf]

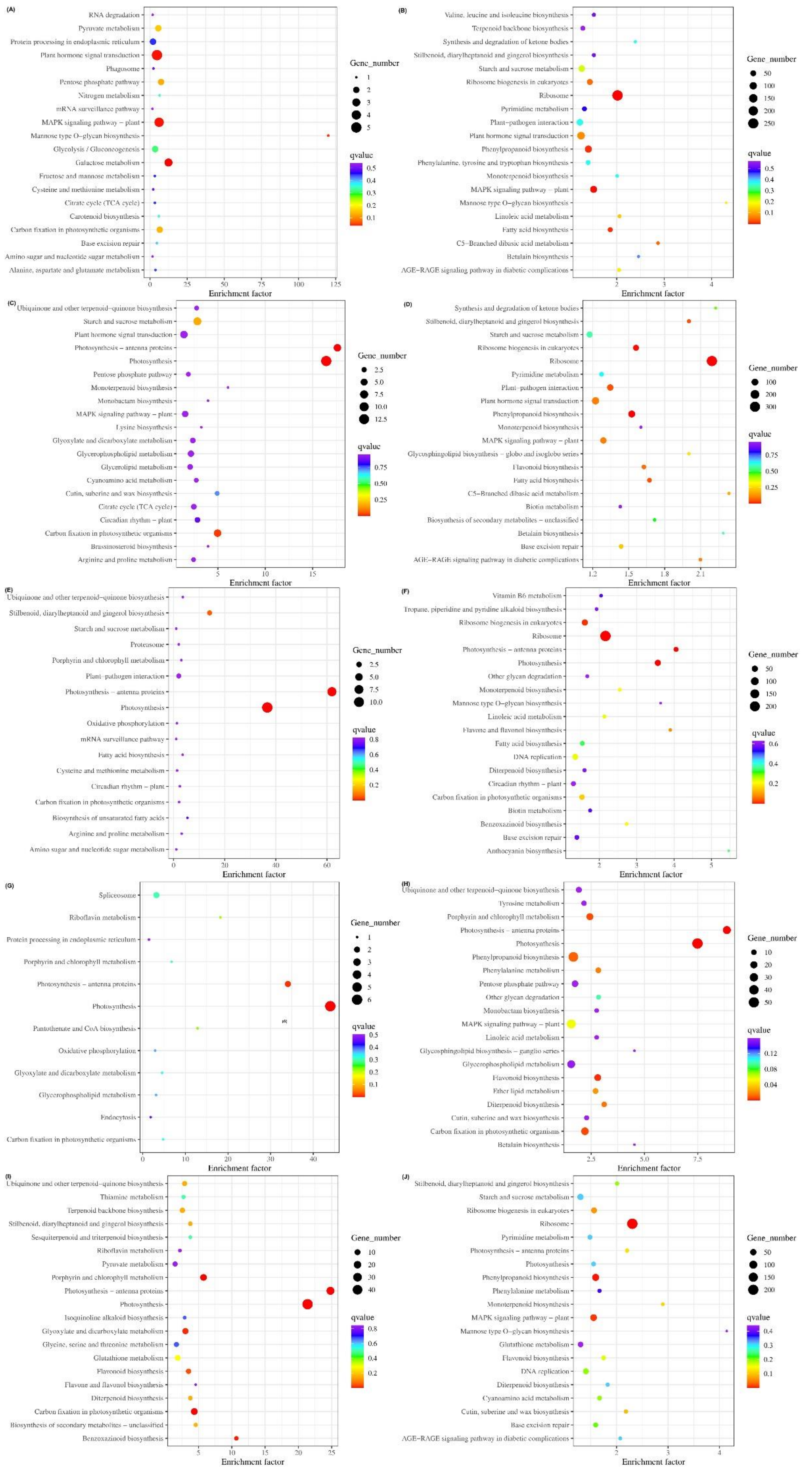
(A)
RNA degradation
Pyruvate metabolism
Protein processing in endoplasmic reticulum
Plant hormone signal transduction
Phagosome
Pentose phosphate pathway
Nitrogen metabolism
mRNA surveillance pathway
MAPK signaling pathway – plant
Mannose type O–glycan biosynthesis
Glycolysis / Gluconeogenesis
Galactose metabolism
Fructose and mannose metabolism
Cysteine and methionine metabolism
Citrate cycle (TCA cycle)
Carotenoid biosynthesis
Carbon fixation in photosynthetic organisms
Base excision repair
Amino sugar and nucleotide sugar metabolism
Alanine, aspartate and glutamate metabolism
0 25 50 75 100 125
Enrichment factor
Gene_number
1 2 3 4 5
qvalue
0.5 0.4 0.3 0.2 0.1
(B)
Valine, leucine and isoleucine biosynthesis
Terpenoid backbone biosynthesis
Synthesis and degradation of ketone bodies
Stilbenoid, diarylheptanoid and gingerol biosynthesis
Starch and sucrose metabolism
Ribosome biogenesis in eukaryotes
Ribosome
Pyrimidine metabolism
Plant–pathogen interaction
Plant hormone signal transduction
Phenylpropanoid biosynthesis
Phenylalanine, tyrosine and tryptophan biosynthesis
Monoterpenoid biosynthesis
MAPK signaling pathway – plant
Mannose type O–glycan biosynthesis
Linoleic acid metabolism
Fatty acid biosynthesis
C5–Branched dibasic acid metabolism
Betalain biosynthesis
AGE–RAGE signaling pathway in diabetic complications
2 3 4
Enrichment factor
Gene_number
50 100 150 200 250
qvalue
0.5 0.4 0.3 0.2 0.1
(C)
Ubiquinone and other terpenoid–quinone biosynthesis
Starch and sucrose metabolism
Plant hormone signal transduction
Photosynthesis – antenna proteins
Photosynthesis
Pentose phosphate pathway
Monoterpenoid biosynthesis
Monobactam biosynthesis
MAPK signaling pathway – plant
Lysine biosynthesis
Glyoxylate and dicarboxylate metabolism
Glycerophospholipid metabolism
Glycerolipid metabolism
Cyanoamino acid metabolism
Cutin, suberine and wax biosynthesis
Citrate cycle (TCA cycle)
Circadian rhythm – plant
Carbon fixation in photosynthetic organisms
Brassinosteroid biosynthesis
Arginine and proline metabolism
5 10 15
Enrichment factor
Gene_number
2.5 5.0 7.5 10.0 12.5
qvalue
0.75 0.50 0.25
(D)
Synthesis and degradation of ketone bodies
Stilbenoid, diarylheptanoid and gingerol biosynthesis
Starch and sucrose metabolism
Ribosome biogenesis in eukaryotes
Ribosome
Pyrimidine metabolism
Plant–pathogen interaction
Plant hormone signal transduction
Phenylpropanoid biosynthesis
Monoterpenoid biosynthesis
MAPK signaling pathway – plant
Glycosphingolipid biosynthesis – globo and isoglobo series
Flavonoid biosynthesis
Fatty acid biosynthesis
C5–Branched dibasic acid metabolism
Biotin metabolism
Biosynthesis of secondary metabolites – unclassified
Betalain biosynthesis
Base excision repair
AGE–RAGE signaling pathway in diabetic complications
1.2 1.5 1.8 2.1
Enrichment factor
Gene_number
100 200 300
qvalue
0.75 0.50 0.25
(E)
Ubiquinone and other terpenoid–quinone biosynthesis
Stilbenoid, diarylheptanoid and gingerol biosynthesis
Starch and sucrose metabolism
Proteasome
Porphyrin and chlorophyll metabolism
Plant–pathogen interaction
Photosynthesis – antenna proteins
Photosynthesis
Oxidative phosphorylation
mRNA surveillance pathway
Fatty acid biosynthesis
Cysteine and methionine metabolism
Circadian rhythm – plant
Carbon fixation in photosynthetic organisms
Biosynthesis of unsaturated fatty acids
Arginine and proline metabolism
Amino sugar and nucleotide sugar metabolism
0 20 40 60
Enrichment factor
Gene_number
2.5 5.0 7.5 10.0
qvalue
0.8 0.6 0.4 0.2
(F)
Vitamin B6 metabolism
Tropane, piperidine and pyridine alkaloid biosynthesis
Ribosome biogenesis in eukaryotes
Ribosome
Photosynthesis – antenna proteins
Photosynthesis
Other glycan degradation
Monoterpenoid biosynthesis
Mannose type O–glycan biosynthesis
Linoleic acid metabolism
Flavone and flavonol biosynthesis
Fatty acid biosynthesis
DNA replication
Diterpenoid biosynthesis
Circadian rhythm – plant
Carbon fixation in photosynthetic organisms
Biotin metabolism
Benzoxazinoid biosynthesis
Base excision repair
Anthocyanin biosynthesis
2 3 4 5
Enrichment factor
Gene_number
50 100 150 200
qvalue
0.6 0.4 0.2
(G)
Spliceosome
Riboflavin metabolism
Protein processing in endoplasmic reticulum
Porphyrin and chlorophyll metabolism
Photosynthesis – antenna proteins
Photosynthesis
Pantothenate and CoA biosynthesis
Oxidative phosphorylation
Glyoxylate and dicarboxylate metabolism
Glycerophospholipid metabolism
Endocytosis
Carbon fixation in photosynthetic organisms
0 10 20 30 40
Enrichment factor
Gene_number
1 2 3 4 5 6
qvalue
0.5 0.4 0.3 0.2 0.1
(H)
Ubiquinone and other terpenoid–quinone biosynthesis
Tyrosine metabolism
Porphyrin and chlorophyll metabolism
Photosynthesis – antenna proteins
Photosynthesis
Phenylpropanoid biosynthesis
Phenylalanine metabolism
Pentose phosphate pathway
Other glycan degradation
Monobactam biosynthesis
MAPK signaling pathway – plant
Linoleic acid metabolism
Glycosphingolipid biosynthesis – ganglio series
Glycerophospholipid metabolism
Flavonoid biosynthesis
Ether lipid metabolism
Diterpenoid biosynthesis
Cutin, suberine and wax biosynthesis
Carbon fixation in photosynthetic organisms
Betalain biosynthesis
2.5 5.0 7.5
Enrichment factor
Gene_number
10 20 30 40 50
qvalue
0.12 0.08 0.04
(I)
Ubiquinone and other terpenoid–quinone biosynthesis
Thiamine metabolism
Terpenoid backbone biosynthesis
Stilbenoid, diarylheptanoid and gingerol biosynthesis
Sesquiterpenoid and triterpenoid biosynthesis
Riboflavin metabolism
Pyruvate metabolism
Porphyrin and chlorophyll metabolism
Photosynthesis – antenna proteins
Photosynthesis
Isoquinoline alkaloid biosynthesis
Glyoxylate and dicarboxylate metabolism
Glycine, serine and threonine metabolism
Glutathione metabolism
Flavonoid biosynthesis
Flavone and flavonol biosynthesis
Diterpenoid biosynthesis
Carbon fixation in photosynthetic organisms
Biosynthesis of secondary metabolites – unclassified
Benzoxazinoid biosynthesis
5 10 15 20 25
Enrichment factor
Gene_number
10 20 30 40
qvalue
0.8 0.6 0.4 0.2
(J)
Stilbenoid, diarylheptanoid and gingerol biosynthesis
Starch and sucrose metabolism
Ribosome biogenesis in eukaryotes
Ribosome
Pyrimidine metabolism
Photosynthesis – antenna proteins
Photosynthesis
Phenylpropanoid biosynthesis
Phenylalanine metabolism
Monoterpenoid biosynthesis
MAPK signaling pathway – plant
Mannose type O–glycan biosynthesis
Glutathione metabolism
Flavonoid biosynthesis
DNA replication
Diterpenoid biosynthesis
Cyanoamino acid metabolism
Cutin, suberine and wax biosynthesis
Base excision repair
AGE–RAGE signaling pathway in diabetic complications
2 3 4
Enrichment factor
Gene_number
50 100 150 200
qvalue
0.4 0.3 0.2 0.1

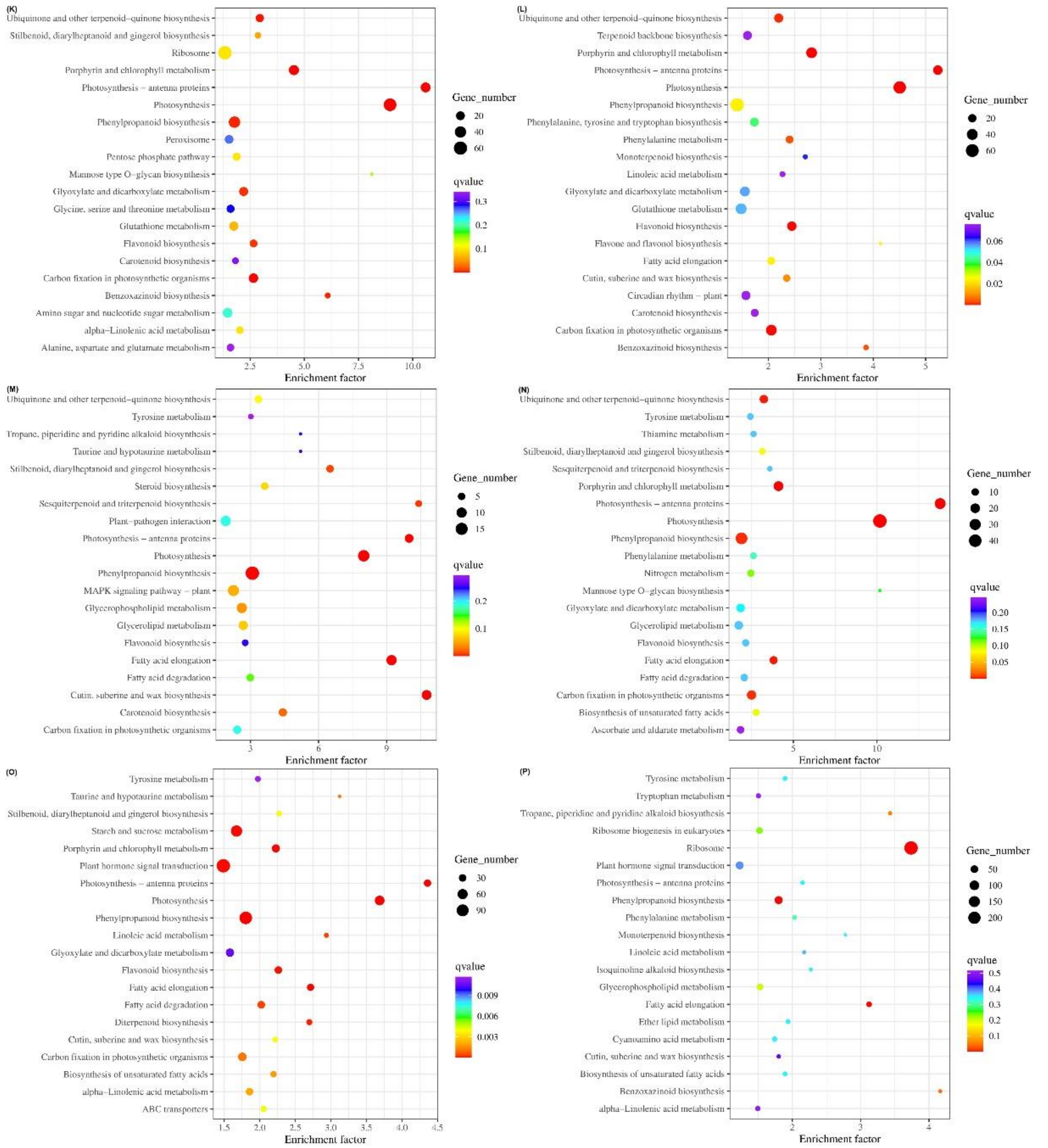

Supplementary Figure 1 20 top KEGG pathways enriched in differentially expressed genes (DEGs) among 16 comparisons, including MES.Red_v_MES.Blue (A), MES.Red_v_MES.White (B), MES.Red_v_MES.Dark (C), MES.Blue_v_MES.White (D), MES.Blue_v_MES.Dark (E), MES.White_v_MES.Dark (F), COL.Red_v_COL.Blue (G), COL.Red_v_COL.White (H), COL.Red_v_COL.Dark (I), COL.Blue_v_COL.White (J), COL.Blue_v_COL.Dark (K), COL.White_v_COL.Dark (L), MES.Red_v_COL.Red (M), MES.Blue_v_COL.Blue (N), MES.White_v_COL.White (O), and MES.Dark_v_COL.Dark (P).
